# Training elite youth soccer players: area per player in small-sided games to replicate the match demands

**DOI:** 10.5114/biolsport.2022.106388

**Published:** 2021-07-28

**Authors:** Andrea Riboli, Sigrid B.H. Olthof, Fabio Esposito, Giuseppe Coratella

**Affiliations:** 1 Department of Biomedical Sciences for Health, Università degli Studi di Milano, Milan, Italy; 2 Research Institute for Sport and Exercise Sciences, Liverpool John Moores University, Liverpool, UK

**Keywords:** Team sports, Football, Performance, Match analysis, Locomotor activities

## Abstract

The aim was to determine the area per player (ApP, m^2^ × player) in small- or large-sided games to replicate the official match demands in elite youth soccer players. Two hundred and twenty-eight players (U15 = 36, U16 = 48, U17 = 49, U18 = 37 and U19 = 58) were monitored during both training (12 183 individual samples) and matches (683 individual samples) across five seasons. Relative (m × min^-1^) total (TD), high-speed running (HSR), very high-speed running (VHSR), sprint and acceleration/deceleration (Acc/Dec) distance were collected. Between-category and between-position comparisons were performed. Area per player was moderately correlated (P < 0.05) with TD (r = 0.401), large (r = 0.621) with HSR, and very largely with VHSR (r = 0.744) and sprint (r = 0.723). An inverse small (r = -0.232; P = 0.039) correlation for Acc/Dec was found. The area per player to replicate the match demands was 158 ± 18, 182 ± 32, 197 ± 37, 212 ± 42 and 156 ± 25 m^2^ × player for TD, HSR, VHSR, sprint and Acc/Dec, respectively. Moderate to very large (ES: 0.79 to 4.66) differences in the area per player across metrics were observed, with sprint > VHSR > HSR > TD = Acc/Dec. Trivial to very large (ES: 0.01 to 2.67) between-category differences in area per player across the same metric were found, with U15 and U16 requiring a larger area per player than other age categories. These findings may help practitioners to recreate the desired external load outcomes with regards to positional match-play demands using specific area per player in small- or large-sided games in youth elite soccer players from U15 to U19.

## INTRODUCTION

In soccer, coaches and sport scientists need to control the training load applied to each player to maximize individual adaptations and improve performance [1, 2]. For this purpose, player-tracking technologies such as global positioning systems are typically utilized to quantify total distance (TD), high-speed running (HSR), very-high speed running (VHSR), sprint and acceleration/deceleration (Acc/Dec) distance during training and matches. This allows the training load to be monitored when using running-based exercises and/or soccer-specific drills such as small- or large-sided games (SSGs) [3].

SSGs are used to improve physical fitness while simultaneously recreating technical, tactical and physical soccer-specific contextual factors [3, 4]. The SSG’s intensity is a crucial feature for practitioners faced with both adult [4] and youth [5] performance development, and practitioners aim to replicate the match demands for player preparation. Comparison of the match vs training loads may help to optimize performance goals [2, 3, 6], conditioning the locomotor activities typically required during the 90-min match demands [2, 3], and/or the most demanding passages of match play [7–9]. In this regard, high-speed running and sprinting play a key role in soccer-specific performance development and injury prevention [10, 11]. Nevertheless, during the training routine, lower exposure to high-speed running or sprinting activities than required during official matches was found in major European soccer leagues such as the English Premier League [12], French Ligue 1 [6], Reserve Spanish La Liga [2], Dutch Eredivisie [13] and Portuguese 1^st^ division [14]. Therefore, an accurate training prescription across different external load exposure (e.g., high-speed running and/or sprinting) may help coaches and sport scientists to optimize top-class performance.

In practice, the manipulation of SSGs may help practitioners to modulate the locomotor activities such as HSR, sprint and/or Acc/Dec distance [3]. Increments in pitch size or reduction in the number of players were found to increase TD, HSR, VHSR and sprint [6]; conversely, when the pitch size is reduced or the number of players is increased, players get more ball touches and the prevalence of the locomotor activities is generally characterized by Acc/Dec [3, 6]. During training interventions using SSGs, the area per player (ApP, expressed as m^2^ × player) was suggested to combine the pitch size and number of players [3, 4, 15]. It is determined as the total pitch area divided by the number of players on the pitch [3, 16]. ApP enables practitioners and researchers to study the effect of the individual player area regardless of team size [3, 15]. It was recently reported that ApP during SSGs was very strongly correlated with the relative (m · min^-1^) TD, HSR and sprint covered, while no correlation for Acc/Dec was found [3]. This highlighted that a larger ApP may increase locomotor demands in elite soccer players [3]. An ApP above 300 m^2^ × player was suggested to induce internal/external load responses near to the individual maximal capacities [17], to replicate official match metabolic and cardiovascular responses [16] and to simulate match tactical behaviours [15]. Thereafter, a minimal ApP of ~311 m^2^ × player to ~316 m^2^ × player was indicated to replicate the high-speed and/or sprint distance relative to official match demands in French Ligue 1 [6] and Italian Serie A soccer players [3].

The SSGs were also pointed out as a useful tool to improve aerobic fitness and technical skills in elite youth soccer players [5]. Similarly to adults, the playing rules, pitch size and number of players seem to affect the external load demands [4]. However, a minimal ApP to replicate the official match demands in elite youth soccer players was not previously investigated. For training prescription purposes, understanding whether or not differences in age may influence the training/match locomotor demands relationship when varying the ApP is hence needed. Therefore, the present study aimed to describe the minimal ApP that could be used to replicate the relative (m × min^-1^) official matches TD, HSR, VHSR, sprint and Acc/Dec using SSGs in youth elite soccer players from U15 to U19. Additionally, the optimal ApP was calculated for each playing position.

## MATERIALS AND METHODS

### Participants

A total sample of 228 elite youth soccer players competing in one of the top-five European youth championship were included in the present study and classified according to their age category as U15 (n = 36), U16 (n = 48), U17 (n = 49), U18 (n = 37) and U19 (n = 58) groups. All participants were classified according to their position: forwards (n = 6, 11, 12, 8 and 13 for U15 to U19, respectively), wide forwards, (n = 4, 3, 5, 3 and 5 for U15 to U19), central midfielders (n = 10, 12, 14, 11 and 14 for U15 to U19), wide midfielders (n = 4, 5, 4, 4 and 11 for U15 to U19), central defenders (n = 7, 11, 8, 6 and 13 for U15 to U19) and wide defenders (n = 5, 6, 6, 5 and 2 for U15 to U19). The goalkeepers were excluded from the data collection. The club’s medical staff certified the health status of each player. An injured player was excluded from data collection for at least one month after their return to full training. The procedures were fully explained to the participants, and to their parents or legal guardian and the club staff. The participants gave their written consent. The local Ethics Committee (protocol #102/14) approved the study that was performed in accordance with the principles of the Declaration of Helsinki (1975) for studies involving human subjects.

### Experimental Design

The present investigation was carried out during the competition period across five seasons (2015–2016 to 2019–2020). The participants undertook their traditional weekly training routine. All sessions were performed on grass or artificial-surface pitches preserved by qualified operators and were conducted at the same time of day to limit the effects of circadian variation. A specialized and highly qualified physician staff recommended and monitored the dietary regime of each player before and after every training session.

A total of 12 183 (504, 818, 4338, 723 and 5800 for U15 to U19, respectively) individual observations with a median of 98 (31, 34, 194, 44 and 186 for U15 to U19, respectively) different formats of SSGs were undertaken. For U15, SSGs ranged from 3 vs 3 to 10 vs 10 with an ApP from 40 m^2^ to 343 m^2^; for U16, SSGs ranged from 2 vs 2 to 10 vs 10 with an ApP from 40 m^2^ to 343 m^2^; for U17, SSGs ranged from 3 vs 3 to 10 vs 10 with an ApP from 50 m^2^ to 286 m^2^; for U18, SSGs ranged from 2 vs 2 to 10 vs 10 with an ApP from 54 m^2^ to 211 m^2^; for U19, SSGs ranged from 3 vs 3 to 10 vs 10 with an ApP from 42 m^2^ to 343 m^2^. Detailed descriptions of SSGs’ characteristics are reported in Supplementary Tables 1, 2, 3, 4 and 5 for U15, U16, U17, U18 and U19, respectively. ApP was calculated excluding the goalkeepers in SSGs. Both small- and large-sided games were abbreviated as SSGs and specified by ApP.

The SSGs were performed under the supervision and motivation of several coaches to keep up a high work rate. For the same reason, a ball was always available by prompt replacement when it went out of play [4]. In SSGs, the corners were replaced by a prompt ball-in-game from the goalkeeper. The SSGs were completed after a standardized 20-min warm-up under the guidance of club staff. A total of 683 individual samples (17, 46, 177, 47 and 396 for U15 to U19) during 116 official matches (3, 10, 30, 10 and 63 for U15 to U19) with a median of 5 ± 6 (range = 40 to 3) individual samples were monitored. The official match pitch size was 105 × 66 m, with a grass surface.

### Procedures

A 10 Hz Global Positioning System unit was used to collect data during both training and matches. Each device was turned on at least 15 min before each session to allow for acquisition of the satellite signal. To reduce the inter-unit differences, each player wore the same unit for every training session over the whole investigation. During both training sessions and matches, TD, HSR (15 to 19.9 km × h^-1^), VHSR (20 to 24 km × h^-1^), sprint (> 24 km × h^-1^) and Acc/Dec (> 3 m × s^-1^) were measured [3]. TD, HSR, VHSR, sprint and Acc/Dec were normalized as relative distance covered in one minute (m·min^-1^) and inserted into the data analysis.

**FIG. 1:**
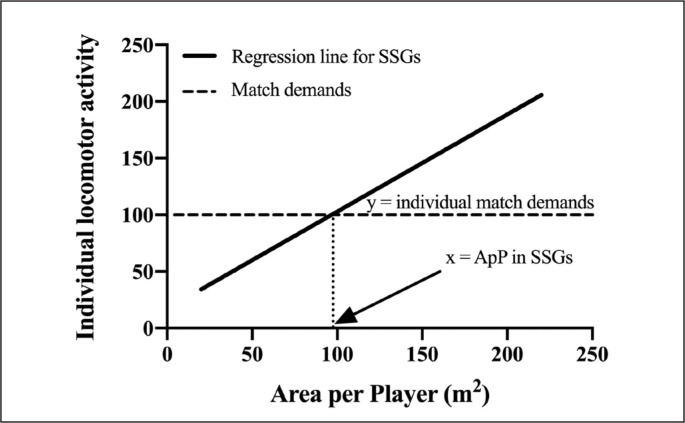
Graphical representation of the procedures used to determine the area per player in SSGs that matches the official match demands. X-axis: the area per player in SSGs; Y-axis: the SSG demands. The regression line shows how the area per player influences the SSG demands. The horizontal dashed line represents the official match demands. From the intersection point of the regression line with the horizontal line (i.e., when the SSG demands equate the official match demands), a vertical dotted line is drawn to the X-axis. The intersection point between the X-axis and the vertical dotted line is the calculated area per player in SSGs necessary to replicate the official match demands.

To determine the ApP that replicates the normalized TD, HSR, VHSR, sprint and Acc/Dec (m × min^-1^) recorded during the official matches across each age category, we first recorded those variables during the official matches. Thereafter, we separately plotted each relationship between ApP and the normalized TD, HSR, VHSR, sprint and Acc/Dec during SSGs. Then, the mean values recorded during the official matches were used to intersect each ApP/ TD, HSR, VHSR, sprint and Acc/Dec relationship recorded in SSGs to calculate the ApP that corresponded to the official match demands (Figure 1), as previously proposed [3].

### Statistical analysis

SPSS (version 26, Chicago, IL, USA) was used to perform the statistical analysis. To check the normal distribution of the sampling, the Shapiro-Wilk test was used. A linear regression analysis was used to calculate the correlation between TD, HSR, VHSR, sprint, and Acc/Dec distance, and the ApP during SSGs. The correlation coefficient was interpreted as follows: *r* = 0.00–0.09 *trivial*, 0.10–0.29 *small*, 0.30–0.49 *moderate*, 0.50–0.69 *large*, 0.70–0.89 *very large*, 0.90–0.99 *nearly perfect*. Thereafter, a linear mixed model analysis was used to calculate the difference in the minimal ApP in TD, HSR, VHSR, sprint and Acc/Dec calculated for each category and position. A post-hoc analysis (Holm-Sidak correction) was used to calculate the differences in the independent factors. Cohen’s *d* effect size with 95% confidence intervals (CI) was used to describe the magnitude of the pairwise differences and interpreted as follows: < 0.20: *trivial*; 0.20–0.59: *small;* 0.60–1.19: *moderate;* 1.20–1.99: *large*; ≥ 2.00: *very large*. Statistical significance was set at α < 0.05. Unless otherwise stated, all values are presented as mean ± standard deviation (SD).

## RESULTS

### Correlations between area per player and locomotor demands

**FIG. 2:**
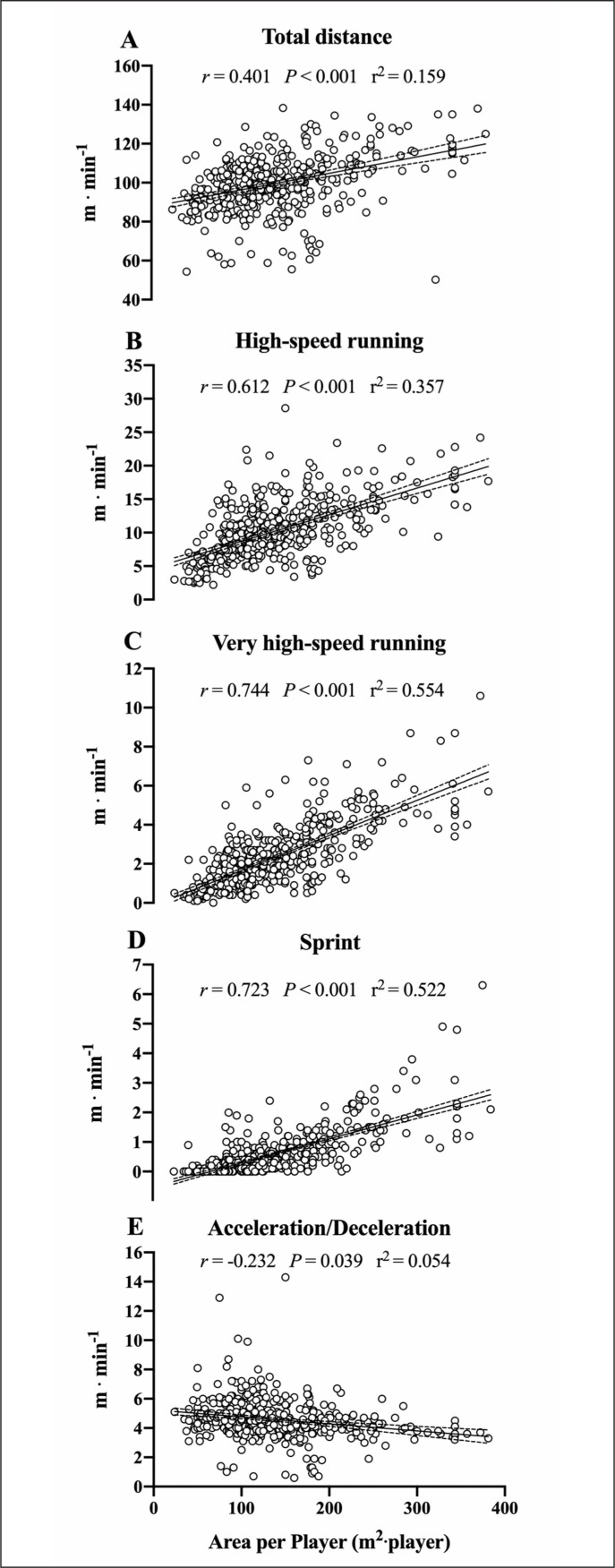
Relationship between area per player (m^2^·player) and relative locomotor demands (m·min^-1^) during small-sided games. The linear regression analysis with 95% confidence interval and the correlation between the area per player and the relative locomotor demands are reported for total distance (Panel A), high-speed running (Panel B), very high-speed running (Panel C), sprint (Panel D) and acceleration/deceleration (Panel E).

The correlations between ApP and locomotor demands for pooled data were *moderate* for TD, *large* for HSR, *very large* for VHSR and sprint and inversely *small* for Acc/Dec (Figure 2). *Moderate* to *very large* correlations for TD, HSR, VHSR and sprint and *small inverse* correlations for Acc/Dec were found across different age categories (Table 1).

**TABLE 1:** Correlations between area per player and locomotor demands during small-sided games for each age category.

	U19			U18			U17			U16			U15		
	r	P		r	P		r	P		r	P		r	P	
TD	**0.723**	**<0.001**	*very large*	**0.362**	**0.029**	*moderate*	**0.362**	**0.023**	*moderate*	**0.406**	**0.017**	*moderate*	**0.619**	**<0.001**	*large*
HSR	**0.838**	**<0.001**	*very large*	**0.356**	**0.022**	*moderate*	**0.311**	**0.019**	*moderate*	**0.732**	**<0.001**	*very large*	**0.759**	**<0.001**	*very large*
VHSR	**0.891**	**<0.001**	*very large*	**0.464**	**0.001**	*moderate*	**0.545**	**<0.001**	*large*	**0.810**	**<0.001**	*very large*	**0.829**	**<0.001**	*very large*
Sprint	**0.843**	**<0.001**	*very large*	**0.335**	**0.026**	*moderate*	**0.664**	**<0.001**	*large*	**0.713**	**<0.001**	*very large*	**0.781**	**<0.001**	*very large*
Acc/Dec	**-0.255**	**0.037**	*small*	-0.142	0.355	*small*	**-0.267**	**0.031**	*small*	-0.243	0.166	*small*	**-0.273**	**0.036**	*small*

Abbreviations: TD: total distance; HSR: high speed running; VHSR: very high-speed running; Acc/Dec: acceleration/deceleration. Bold text highlights significant correlations.

### Area per player to replicate official match demands using SSGs

**FIG. 3:**
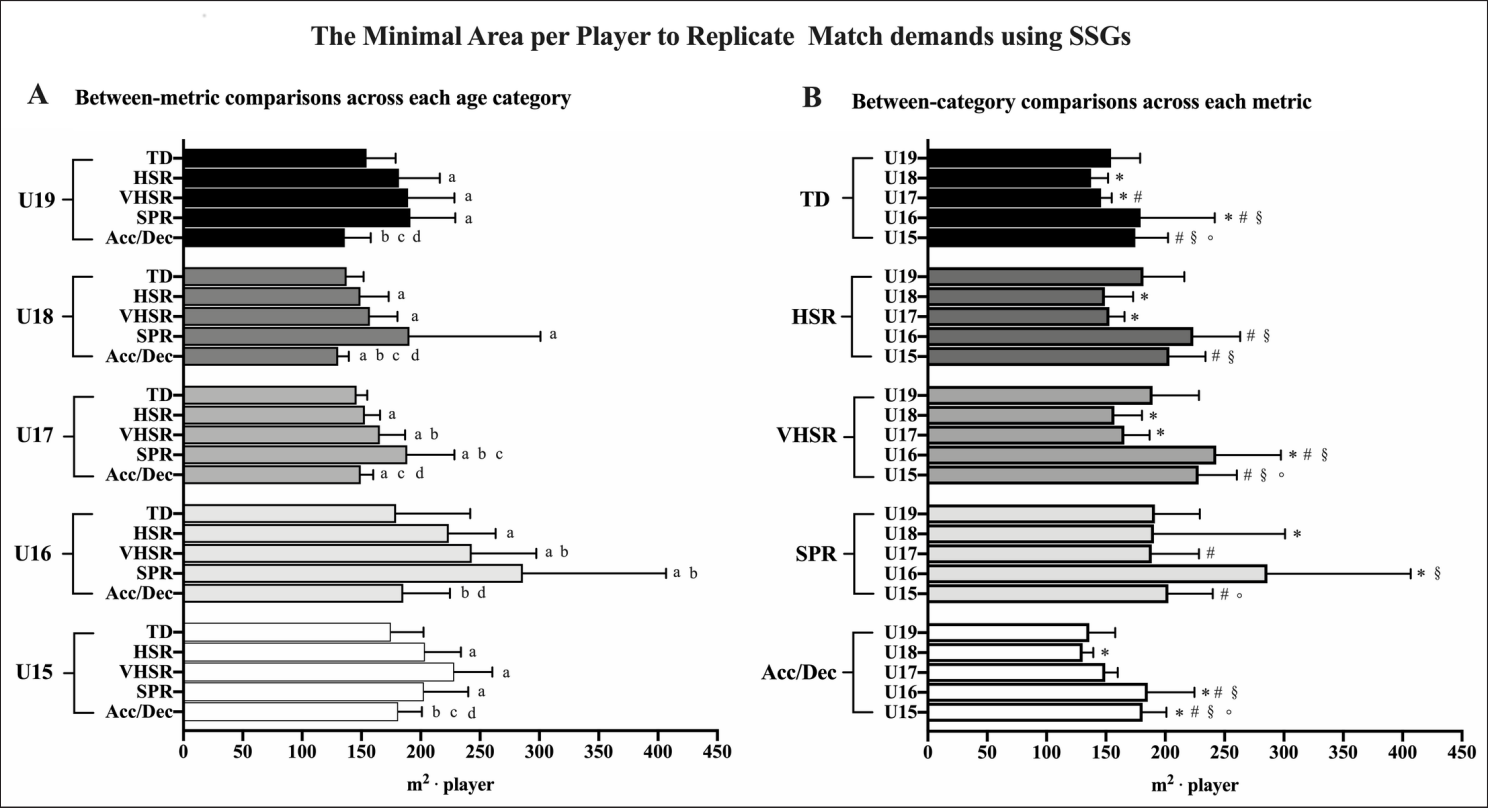
Minimal area per player (m^2^·player) to replicate the locomotor match demands (m·min^-1^) using small-sided games across different age categories. Between-metric comparisons for ApP within the same age category (Panel A) and between-category comparisons for ApP within the same metric (Panel B) are shown. Data are reported as mean (SD). TD: total distance; HSR: high-speed running; VHSR: very high-speed running; SPR: sprint; Acc/Dec: acceleration/deceleration. For Panel A: ^a^*P* < 0.05 vs TD; ^b^*P* < 0.05 vs HSR; ^c^*P* < 0.05 vs VHSR; ^d^*P* < 0.05 vs SPR. For Panel B: ^*^P < 0.05 vs U19; ^#^*P* < 0.05 vs U18; ^§^*P* < 0.05 vs U17; °*P* < 0.05 vs U16.

For pooled data, sprint showed *slightly* (ES: 0.38; CI: 0.18 to 0.57) higher (*P* = 0.041) ApP than VHSR, *moderately* (ES: 0.79; CI: 0.59 to 0.99) higher (*P* = 0.009) ApP than HSR and *much* higher (*P* < 0.001) ApP than TD (ES: 1.65; CI: 1.43 to 1.87). VHSR showed *slightly* higher (*P* = 0.009) ApP than HSR (ES: 0.41; CI: 0.22 to 0.61) and *much* higher (*P* < 0.001) ApP than TD (ES: 4.66; CI: 4.29 to 5.03). HSR showed a *moderately* higher (*P* < 0.001) ApP than TD (ES: 0.91; CI: 0.71 to 1.11). The ApP for Acc/Dec was *moderately* to *largely* lower (*P* < 0.001) than HSR (ES: -0.89; CI: -1.09 to 0.69), VHSR (ES: -1.25; CI: -1.46 to -1.04) and sprint (ES: -1.60; CI: -1.82 to -1.37); no difference (*P* > 0.05) in ApP between Acc/Dec and TD was found. Detailed ApP descriptions for each metric across difference categories are reported in Figure 3 (Panel A).

As reported in Figure 3 (Panel B), between-category differences in ApP across the same metric were *trivial* to *large* (ES: 0.08 to 1.67) for TD, *trivial* to *very large* (ES: 0.19 to 2.51) for HSR, *small* to *very large* (ES: 0.30 to 2.67) for VHSR, *trivial* to *moderate* (ES: 0.01 to 1.10) for sprint and *trivial* to *very large* (ES: 0.12 to 3.75) for Acc/Dec.

### Area per player to replicate official match demands using SSGs across positions

**FIG. 4:**
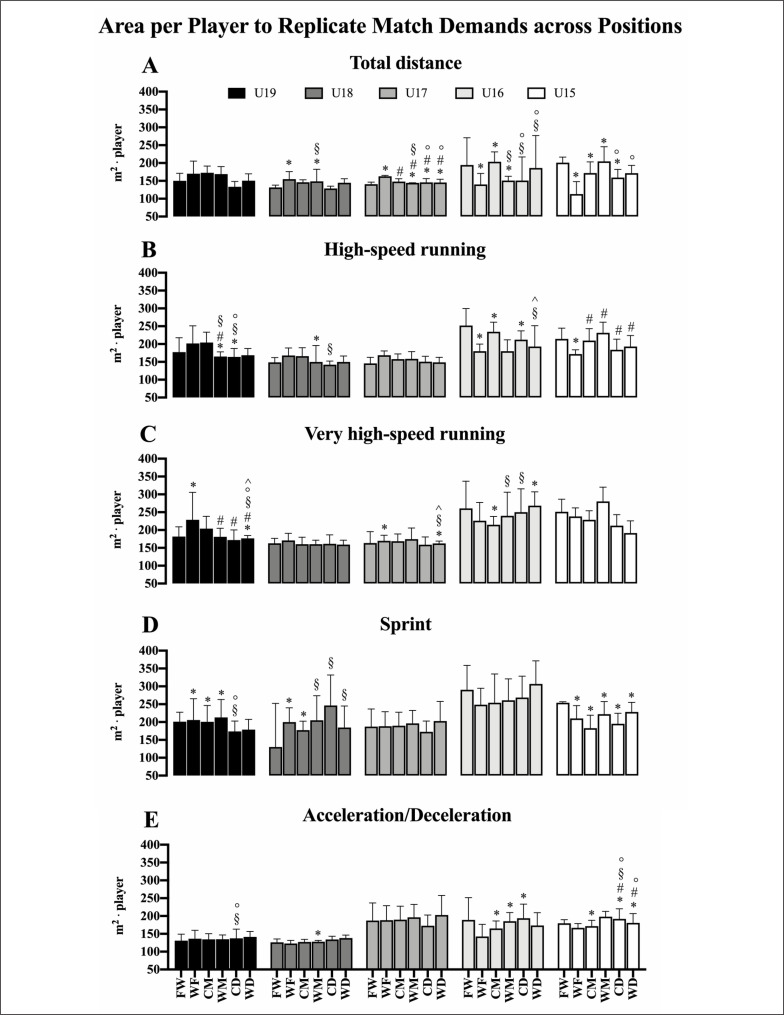
Area per player (m^2^·player) to replicate the locomotor match demands using small-sided games for different age categories across different positions is shown. Total distance (Panel **A**), high-speed running (Panel **B**), very high-speed running (Panel **C**), sprint (Panel **D**) and acceleration/Deceleration (Panel **E**). Data are reported as mean (SD). FW: forwards; WF: wide forwards; CM: central midfielders; WM: wide midfielders; CD: central defenders; WD: wide defenders. ^*^P < 0.05 vs FW; ^#^*P* < 0.05 vs WF; ^§^*P* < 0.05 vs CM; °*P* < 0.05 vs WM; ^*P* < 0.05 vs CD.

Figure 4 shows the minimal ApP to replicate match demands for TD, HSR, VHSR, sprint and Acc/Dec across different positions for each age category.

## DISCUSSION

This study aimed to investigate the optimal ApP to mimic the physical match demands across youth teams and positions. The main finding was a detailed calculation of the ApP in SSGs necessary to replicate the TD, HSR, VHSR, sprint and Acc/Dec recorded during the official matches in youth elite soccer players from U15 to U19. A higher ApP increased TD, HSR, VHSR and sprint across each age category. Conversely, Acc/Dec showed only a *small* inverse correlation with ApP in U15, U17 and U19, while no effects of ApP on Acc/Dec for U16 and U18 were observed. Moreover, the higher the speed threshold was, the larger was the ApP required (i.e., sprint > VHSR > HSR > TD = Acc/Dec). Some between-category (i.e., U15 to U19) differences in ApP to overload official match demands within the same metric (i.e., TD, HSR, VHSR, sprint, Acc/Dec) were found, with U15 and U16 apparently requiring larger ApP. Lastly, the ApP to overload match demands for each position across different categories were provided. The current results highlighted that an individualized approach for each category and position is required to replicate the match demands in elite youth soccer players.

The total high-intensity running distance [18] is indicated as a key factor for success in soccer match performance in addition to the technical skills to maintain greater ball possession [19], the total distance covered with ball possession [20], and the tactical behaviours [21]. Within the weekly training routines, SSGs are predominantly used to elicit high-intensity running [4], as technical drills with or without ball possession [9], and to improve tactical behaviours [21]. Interestingly, SSGs were previously shown to lead to similar enhancement in aerobic fitness as high-intensity interval training running [22]. Therefore, conditioning through sport-specific drills can be a successful option to recreate soccer-specific contextual factors that are typically required during official match performance [3, 4]. The present findings showed that higher ApP increased locomotor demands in different categories from U15 to U19 across each metric, especially for HSR, VHSR, and sprint. This implied that larger ApP allowed for reaching higher locomotor loads, since more space was needed to reach high-speed running. Conversely, Acc/Dec showed only a *small* inverse correlation with ApP for U15, U17 and U19, while no effect of ApP on Acc/Dec was found for U16 and U18. Given the reduced space with smaller ApP, which required continuous accelerating and decelerating activities, a clear relationship was hard to find. The present results were in line with findings for elite adult Serie A soccer players [3]. An increment in the ApP used during SSGs is recommended when practitioners aim to increase the locomotor demands to condition youth soccer players properly.

The current study used a novel approach [3] to model a specific ApP to recreate the official match demands for TD, HSR, VHSR, sprint, and Acc/Dec in youth soccer players. The results showed that a larger ApP was necessary to recreate HSR (~182 m^2^·player), VHSR (~197 m^2^·player), and sprint (~212 m^2^·player) compared to TD (~158 m^2^·player) and Acc/Dec (~156 m^2^·player). Those data put emphasis on the ApP ~200 m^2^·player or more, necessary to replicate very high-intensity activities. In a similar elite academy population, it was previously reported that an ApP of ~300 to ~320 m^2^ × player was needed to induce internal/external load responses near to the individual maximal capacities [17] and/or to replicate the official match metabolic and cardiovascular responses [16]. Additionally, a tactical analysis during SSGs performed in U13 to U19 soccer players highlighted that interpersonal distances, team length and team width increased near to the match demands when incrementing the number of players (i.e., from 4 vs 4 to 8 vs 8) within the same ApP (i.e., ~320 m^2^ × player) [15]. However, the present findings for the first time provided a detailed ApP calculation to recreate locomotor demands using SSGs in youth soccer players. Comparing the results with adults, in French Ligue 1 soccer players, a minimal ApP ~311 m^2^ × player was indicated to replicate the high-speed running distance relative to official match demands [6]. Similarly, ApP ~316 m^2^ × player was shown to replicate the sprinting activities in Italian Serie A soccer players [3]. Additionally, playing SSGs in an ApP of ~320 m^2^ × player was also reported as useful to recreate the tactical variability for attacking exploration and defending organization [23]. Therefore, the ApP is a useful tool to condition the physiological responses [17], external load demands [3] and tactical behaviours [23] with regards to match demands both in youth and adult elite soccer players.

Current findings modelled an ApP of ~200 m^2^·player as optimal pitch dimensions to replicate physical match demands. This is slightly lower compared to the recommendations of previous studies [3, 6, 17]. The present youth elite soccer players may have different anthropometric, physiological and technical characteristics, as well as different coaching style and tactical behaviours that may underly a lower ApP (i.e. ~200 m^2^·player) than adult elite soccer players (i.e. ~300 m^2^·player). As such, for replication purposes an individualized approach is required due to the typical soccer-specific variability in athletes’ characteristics, coaches’ style of play, etc., possibly affecting ApP calculation. However, ApP of ~200 to ~300 m^2^·player is larger than ApP previously utilized for SSGs [4, 24]. As a mere example, two reviews on SSG demands reported a usual ApP of ~91 m^2^·player (i.e. ranging from ~25 to ~200 m^2^·player) [4] or ~93 m^2^·player (i.e. ranging from ~25 to ~273 m^2^·player) [24]. However, neither of those studies compared SSG demands with match requirements to suggest an optimal ApP. Therefore, despite soccer-specific intrinsic between-group variability, coaches and sport scientists could consider ~200 to ~300 m^2^·player to properly replicate very-high speed to sprint activities using SSGs when required.

Furthermore, the current findings indicated that a tailored ApP is necessary in U15 to U19 players to replicate the official match demands across the different metrics. Overall, larger ApP was needed to recreate more intense efforts in all categories. Interestingly, U15 was the only category that showed larger ApP to replicate VHSR than sprint, in contrast with the overall results. This could be possibly due to the fixed speed thresholds used in the current study, which were not based on the individual maximal sprint ability, as suggested to overcome such an issue [25]. It is indeed possible that the > 24 km·h^-1^ speed threshold for the sprint zone could be too high to accurately determine the distance covered in U15 soccer players. Overall, greater ApP is needed for each metric in U15 and U16 compared to the remaining age categories. Although surprising at first glance, it is possible that the older and possibly physically stronger players might need less space to reach the high-speed thresholds and enter the high-speed zones. When practising SSGs, coaches could thus manipulate the ApP depending on the purposes of each session [3]. Remarkably, since the average match demands could fail to fully account for the actual peak demands [7, 9] and the distribution of the maximal intensities [8] that occur during official matches, SSGs may be used to recreate the most demanding passages of match play across different time durations, with or without ball possession as previously reported [3, 9].

The present findings also showed different ApP across playing positions within the same age category. The lack of consistency in the between-position differences in ApP across the age categories suggests that an individualized approach is necessary. As such, a first suggestion might be to group the players by position when practising SSGs. However, this does not recreate the contextual factors characterizing the matches given the homogeneous tactical characteristics of the players involved. Therefore, together with the traditional SSG format involving more positions simultaneously, additional positional SSG drills and/or running-based exercises may be included. For example, adjunctive high-intensity activities (e.g., sprinting-based exercises) during or after SSGs can be utilized to overload some positions and/or individual players, when necessary.

The present study has some limitations. Firstly, the internal load parameters (e.g. heart rate) and the rate of perceived exertion were not examined, and we acknowledge that should be coupled with the external load metrics to describe accurately the match demands. However, some technological limitations (e.g., the use of portable thoracic bands especially during official matches) or some contextual limitations (e.g., athletes buy-in to collect rate of perceived exertion after each SSG format) can affect the possibilities to monitor consistently both the internal and perceived load for the aim of the present study. Secondly, individualizing the speed thresholds (based on individual maximal sprint ability) and/or increasing sample size for each position may help to further improve the understanding of the between-category and between-position differences. Thirdly, the maturity status can affect the physical and anthropometric characteristics of youth soccer players within the same age category and should be taken into account [26]. Lastly, for replication purposes an individualized approach is required due to the typical soccer-specific variability (athletes’ characteristics, coaches’ style of play, etc.) possibly affecting ApP calculation.

The present findings have a number of practical applications. In the first instance, the specific ApP can be used in SSGs to replicate, underload or overload the match demands in youth soccer players, as also previously suggested for elite adults players [3]. Having established that larger ApP led to higher locomotor demands, a minimum of ~200 m^2^ × player seemed to be required to properly stimulate the high-speed activities in youth players. For this purpose, coaches could modify the number of players and/or the pitch size during SSGs to focus on specific metrics. However, since smaller ApP are used in practice to stimulate technical activities (e.g., ball touches, shots, crosses), additional rules and/or supplementary exercises such as running-based exercises individualized on the cardiorespiratory and metabolic capacity [27, 28] and/or soccer-specific drills to overload the most demanding phases of match play [9] properly based on the distribution of match activities [8] may be recommended. Moreover, younger players (i.e., U15 and U16) appear to require larger ApP for each metric, probably due to an incomplete maturity status that precludes them from greater acceleration capacity to reach high-speed zones in a larger space. Additionally, the inconsistency in positional differences suggested that different positions may be overload or underload within the same ApP. Therefore, some positions may need supplementary activities. Lastly, it should be remarked that soccer-specific drills only may not sufficiently prepare players for the match demands [29]. This may be due to the individual capacity that may exceed the actual stimuli received for some positions (e.g., central defenders). As such, individual physiological (e.g., hear rate) and psychophysiological (e.g., rate of perceived exertion) responses should be collected together with the external load metrics [1]. In case of an insufficient stimulus, submaximal and/or maximal individual-based exercises should be included [30].

### Practical Applications

Larger ApP should be used to increase TD, HSR, VHSR and sprint, while Acc/Dec is less affected by ApP manipulation.

A minimum of ~200 m^2^ × player seems necessary to properly stimulate the high speed and sprint activities in youth players.

The younger players (i.e., U15 and U16) appear to require larger ApP (~230 m^2^ × player) due to possibly lower acceleration capacity (i.e. lower maturity status).

When a specific large ApP is not feasible, supplementary training prescriptions using SSGs with adjunctive rules, running-based exercises and/or positional drills seem to be required to effectively overload each player.

## CONCLUSIONS

In conclusion, the present study showed positive correlations between ApP and the external load metrics in youth elite soccer players from U15 to U19. With the exception of Acc/Dec, greater ApP induced higher locomotor demands for TD, HSR, VHSR and sprint. A minimal ApP during SSGs to replicate or overload the match demands for each metric was suggested. Moreover, greater ApP is needed for each metrics in U15 and U16 compared to the other age categories. These findings may help practitioners to recreate the desired external load outcomes with regards to positional match-play demands using specific area per player in small- or large-sided games in youth elite soccer players from U15 to U19.

## Figures and Tables

**Supplementary table 1:** Pitch size and locomotor demands during small-sided games in U15 soccer players.

N° Players	m	m^2^	m^2^ · player	TD	HSR	VHSR	SPR	Acc/Dec
**10 vs 10**	45 × 60	2700	135	101.4 (8.9)	10.2 (3.2)	1.7 (1.2)	0.3 (0.5)	4.2 (1.0)
	40 × 60	2400	120	98.9 (7.0)	8.0 (1.4)	1.5 (1.1)	0.2 (0.2)	4.6 (0.5)
	45 × 55	2475	124	98.6 (8.3)	8.3 (3.1)	1.5 (1.2)	0.3 (0.3)	5.0 (1.2)
	50 × 60	3000	150	120.1 (9.6)	14.9 (4.5)	2.8 (1.8)	0.5 (0.7)	4.4 (0.8)
	50 × 68	3400	170	101.0 (9.9)	10.0 (2.9)	1.9 (1.8)	1.0 (2.0)	4.6 (1.3)
	65 × 60	3900	195	102.9 (11.0)	9.5 (4.1)	2.0 (1.5)	0.3 (0.4)	4.5 (0.8)
	66 × 104	6864	343	119.6 (9.0)	19.3 (3.5)	5.2 (2.4)	1.3 (1.7)	4.1 (1.1)
	50 × 80	4000	200	112.4 (10.0)	16.9 (4.0)	4.1 (2.2)	1.3 (1.2)	4.1 (1.0)
	60 × 104	6240	312	107.3 (8.4)	15.8 (3.6)	4.5 (2.4)	1.1 (1.8)	3.7 (0.6)
	60 × 100	6000	300	115.8 (4.5)	17.5 (1.9)	4.6 (1.7)	2.0 (0.9)	3.8 (0.8)

**9 vs 9**	35 × 65	2275	126	113.2 (8.9)	13.8 (3.4)	2.1 (1.3)	0.4 (0.5)	4.9 (1.1)
	66 × 52	3432	191	110.4 (10.7)	13.8 (3.3)	3.1 (1.3)	1.2 (1.1)	4.7 (0.9)
	45 × 60	2700	150	100.1 (6.8)	8.1 (4.1)	1.1 (1.3)	0.2 (0.7)	2.9 (0.6)
	50 × 60	3000	167	97.0 (7.1)	8.9 (4.1)	1.7 (1.4)	0.1 (0.3)	3.3 (0.9)
	65 × 50	3250	181	105.5 (8.2)	11.4 (3.7)	2.1 (1.4)	0.3 (0.4)	4.2 (0.9)

**8 vs 8**	40 × 50	2000	125	103.4 (9.8)	12.5 (2.7)	2.5 (1.1)	0.2 (0.3)	3.8 (0.7)

**7 vs 7**	40 × 50	2000	143	105.9 (8.9)	12.0 (4.7)	1.9 (1.7)	0.2 (0.5)	4.2 (1.0)
	40 × 52	2080	149	104.6 (8.9)	10.4 (3.0)	1.9 (1.4)	0.3 (0.6)	4.9 (0.9)
	65 × 50	3250	232	120.6 (17.0)	11.4 (6.1)	2.1 (2.1)	0.3 (1.0)	4.2 (1.9)
	30 × 50	1500	107	88.8 (10.5)	4.9 (3.0)	0.4 (0.7)	0.0 (0.0)	5.1 (1.6)

**6 vs 6**	30 × 45	1350	113	81.3 (7.5)	4.7 (2.2)	1.2 (1.1)	0.1 (0.3)	3.1 (0.9)
	22 × 22	484	40	80.7 (8.8)	2.7 (1.7)	0.2 (0.3)	0.0 (0.0)	4.5 (1.1)

**5 vs 5**	27 × 30	810	81	93.1 (9.9)	7.6 (3.8)	0.5 (1.3)	0.0 (0.1)	4.5 (1.1)

**4 vs 4**	22 × 30	660	83	93.6 (8.3)	7.4 (4.5)	0.5 (1.0)	0.0 (0.0)	5.0 (1.6)
	30 × 52	1560	195	108.0 (8.8)	15.9 (4.6)	3.8 (2.1)	0.6 (0.8)	5.2 (0.8)
	50 × 35	1750	219	93.6 (16.2)	8.1 (2.2)	1.2 (1.1)	0.1 (0.3)	4.7 (0.9)

**3 vs 3**	20 × 30	600	100	97.3 (5.9)	7.4 (3.1)	0.7 (0.8)	0.0 (0.0)	6.0 (1.6)
	25 × 30	750	125	96.4 (9.0)	7.6 (3.3)	0.6 (0.7)	0.0 (0.0)	6.3 (1.5)
	18 × 22	396	66	83.3 (10.1)	6.4 (3.9)	1.0 (1.4)	0.2 (0.2)	5.0 (1.9)

The small-sided games are split for the number of players and pitch size (width × length). The total pitch area (m^2^) and area per player (m^2^·player) have been calculated. Average locomotor demands are reported for total distance (TD), high-speed running (HSR), very high-speed running (VHSR), sprint (SPR) and acceleration/Deceleration (Acc/Dec). Data are reported as mean (SD).

**Supplementary table 2:** Pith size and locomotor demands during small-sided games in U16 soccer players.

**N° Players**	**m**	**m** ^2^	**m**^2^ **× player**	**TD**	**HSR**	**VHSR**	**SPR**	**Acc/Dec**

**10 vs 10**	66 × 104	6864	343.2	115.6 (12.8)	16.7 (5.4)	4.8 (2.1)	1.8 (1.7)	4.5 (1.1)
	66 × 70	4620	231	103.7 (11.4)	12.0 (4.0)	3.0 (2.1)	0.7 (1.2)	4.1 (1.1)
	65 × 70	4550	227.5	90.2 (9.0)	8.0 (2.9)	2.4 (1.4)	0.6 (0.8)	4.9 (0.6)
	52x66	3432	171.6	101.2 (10.9)	10.7 (3.3)	2.5 (1.6)	0.4 (0.5)	4.2 (1.1)
	40 × 60	2400	120	83.9 (11.5)	6.6 (3.5)	1.3 (1.7)	0.2 (0.5)	3.8 (1.3)
	45 × 52	2340	117	87.4 (8.2)	8.3 (3.5)	2.0 (1.4)	0.1 (0.1)	3.9 (1.1)
	40 × 52	2080	104	83.0 (16.4)	6.8 (4.1)	1.0 (0.7)	0.1 (0.2)	3.5 (1.7)
	40 × 50	2000	100	92.0 (12.3)	7.5 (3.6)	1.3 (1.1)	0.1 (0.1)	4.6 (1.4)

**10 vs 10 (+1f)**	60x48	2880	137.1	79.8 (13.2)	6.1 (3.8)	1.1 (0.9)	0.1 (0.2)	3.4 (2.0)

**9 vs 9**	52 × 40	2080	115.5	92.4 (12.3)	9.2 (4.3)	1.7 (1.0)	0.2 (0.6)	4.5 (1.2)
	52 × 60	2400	133.3	112.7 (12.9)	15.4 (5.4)	4.4 (2.4)	1.0 (0.9)	4.7 (1.1)
	60 × 70	4200	233.3	124.1 (13.8)	19.3 (5.5)	5.7 (3.2)	2.2 (3.6)	4.3 (1.1)
	66 × 70	4620	256.7	113.4 (11.6)	14.8 (4.0)	4.2 (1.7)	1.0 (0.8)	4.3 (0.8)
**9 vs 8**	24 × 43	1032	60.7	75.2 (11.6)	3.1 (2.2)	0.3 (0.4)	0.0 (0.1)	4.1 (1.1)

**8 vs 8**	40 × 50	2000	125	96.4 (10.6)	9.7 (3.8)	1.9 (1.4)	0.4 (0.4)	4.6 (1.2)
	52 × 66	3432	214.5	109.4 (14.4)	14.4 (5.3)	3.7 (2.0)	0.8 (0.9)	4.5 (1.0)

**8 vs 8 (+1f)**	45x40	1800	105.8	90.6 (9.4)	7.6 (4.7)	0.6 (0.7)	0.0 (0.0)	3.8 (1.2)

**7 vs 7**	52x40	2080	148.6	100.2 (8.3)	10.0 (2.7)	2.3 (1.3)	0.3 (0.3)	5.1 (0.9)
	50x40	2000	142.9	93.8 (9.3)	7.6 (1.7)	1.4 (0.7)	0.2 (0.2)	5.0 (0.9)

**5 vs 5**	25x18	450	45	85.1 (14.4)	5.5 (2.8)	0.8 (0.6)	0.2 (0.3)	5.9 (2.7)
	35x30	1050	105	80.5 (7.1)	5.0 (1.6)	0.2 (0.1)	0.0 (0.0)	6.2 (1.2)
	36x30	1080	108	90.1 (16.2)	11.5 (5.3)	2.1 (1.7)	0.4 (0.5)	3.4 (1.9)
	30x45	1350	135	96.7 (16.6)	10.7 (4.0)	1.4 (1.2)	0.1 (0.4)	4.7 (1.3)

**4 vs 3**	25x30	750	107.1	84.1 (7.6)	6.6 (1.5)	0.3 (0.4)	0.0 (0.0)	4.9 (0.7)

**4 vs 4 (+2f)**	20x25	500	50	63.7 (18.5)	2.2 (1.6)	0.0 (0.0)	0.0 (0.0)	5.7 (2.1)

**4 vs 4 (+3f)** **4 vs 4**	35x30	1050	95.5	63.7 (18.5)	2.2 (1.6)	0.0 (0.0)	0.0 (0.0)	5.7 (2.1)
	30x40	1200	150	95.4 (7.2)	11.3 (4.3)	2.2 (0.9)	0.4 (0.9)	5.6 (0.7)
	25x35	875	109.4	95.9 (10.9)	8.3 (2.8)	0.9 (0.8)	0.1 (0.2)	5.4 (1.2)

**3 vs 3**	24x32	768	128	99.3 (6.7)	9.7 (3.1)	1.0 (3.1)	0.1 (0.7)	6.1 (0.2)

**3 vs 3 (+1f)**	25x30	750	125	77.7 (6.3)	5.1 (2.5)	0.9 (0.3)	0.0 (0.0)	4.9 (1.0)

**2 vs 2**	10x16	160	40	54.4 (8.2)	4.2 (1.5)	2.2 (1.2)	0.9 (1.1)	3.1 (1.0)
	32x40	1280	320	50.2 (12.5)	9.4 (3.7)	3.8 (2.6)	0.8 (0.9)	3.8 (1.5)

The small-sided games are split for the number of players and pitch size (width × length). The total pitch area (m^2^) and area per player (m^2^ · player) have been calculated. The number of floaters, when required, is reported between bracket (i.e., one floater: +1f, two floaters: +2f, etc.). Average locomotor demands are reported for total distance (TD), high-speed running (HSR), very high-speed running (VHSR), sprint (SPR) and acceleration/Deceleration (Acc/Dec). Data are reported as mean (SD).

**Supplementary table 3:** Pith size and locomotor demands during small-sided games in U17 soccer players.

N° Players	m	m^2^	m^2^⋅player	TD	HSR	VHSR	SPR	Acc/Dec
10 *vs* 10	45 × 40	1800	90	97.1 (17.8)	6.4 (3.5)	0.4 (0.5)	0.2 (0.5)	4.8 (1.4)
	40 × 40	1600	160	106.2 (16.5)	10.9 (5.7)	2.0 (1.6)	0.3 (0.5)	5.0 (1.3)
	60 × 52	3120	156	87.6 (15.3)	9.5 (4.3)	2.1 (1.6)	0.8 (1.1)	4.0 (1.3)
	66 × 52	3432	172	84.1 (24.7)	8.3 (5.3)	2.3 (2.0)	0.6 (0.8)	3.2 (2.1)
	66 × 60	3960	198	93.2 (10.3)	10.3 (3.9)	2.5 (2.4)	0.5 (0.9)	3.7 (0.8)
	65 × 62	4030	201	96.0 (13.0)	10.5 (4.2)	2.8 (2.6)	0.7 (0.6)	3.7 (1.0)
	70 × 66	4620	231	102.2 (8.2)	10.9 (2.8)	3.3 (1.2)	1.5 (1.2)	3.7 (0.7)
	75 × 66	4950	247	104.6 (10.l)	13.4 (3.8)	3.8 (1.4)	1.5 (1.1)	3.8 (0.9)
	105 × 68	7140	357	111.5 (11.1)	13.8 (3.3)	4.0 (1.8)	1.2 (0.9)	3.6 (0.9)
	66 × 50	3300	165	84.5 (18.8)	8.4 (4.5)	2.1 (1.6)	0.6 (0.8)	3.1 (1.9)
	66 × 64	4224	211.2	101.9 (14.2)	13.0 (5.6)	4.3(2.3)	1.4 (1.5)	4.8 (0.7)
	66 × 40	2640	132	78.8 (23.2)	7.0 (4.8)	1.8 (1.6)	0.6 (0.7)	2.6 (2.5)
	104 × 66	6864	343	114.9 (11.7)	16.5 (6.0)	4.5 (1.8)	2.2 (1.7)	4.1 (0.7)

10 *vs* 10 (+1f)	45 × 35	1575	75	90.4 (14.5)	7.8 (6.1)	1.6 (1.3)	0.3 (0.6)	4.4 (1.3)
	54 × 36	1944	92.6	85.2 (8.5)	8.0 (3.9)	1.4 (0.8)	0.3 (0.4)	4.2 (1.0)
	66 × 47	3102	148	104.9(12.9)	16.0 (4.2)	3.6 (2.2)	1.0 (1.3)	3.9 (0.5)
	60 × 50	3000	143	99.2 (9.4)	10.6 (3.7)	2.7 (1.0)	0.8 (0.9)	3.5 (1.0)
	66 × 50	3300	157	55.5 (12.4)	3.4 (2.4)	0.9 (1.0)	0.2 (0.5)	0.6 (0.9)
	66 × 51	3366	160	106.3 (15.6)	13.2 (5.1)	3.4 (2.1)	1.0 (1.0)	4.2 (1.1)
	66 × 52	3432	163	90.0 (10.1)	8.1 (3.9)	2.1 (1.6)	0.5 (0.7)	3.3 (1.0)
	66 × 55	3630	173	73.9 (22.9)	5.7 (5.2)	1.3 (1.8)	0.4 (0.8)	1.9 (1.9)
	66 × 61	4026	192	101.8 (17.6)	13.1 (7.7)	3.3 (2.7)	0.8 (1.2)	4.5 (1.1)
	72 × 66	4752	226	112.4 (14.0)	15.1 (4.5)	4.9 (2.9)	2.3 (1.6)	4.8 (1.3)
	78 × 66	5148	245	84.8 (23.7)	10.6 (6.2)	3.1 (1.9)	1.5 (1.8)	1.9 (1.8)

10 *vs* 10 (+2f)	66 × 61	4026	183	65.3 (21.0)	4.6 (2.3)	1.1 (1.4)	0.5 (0.9)	1.0 (1.3)
	47 × 40	1880	85.5	111.5 (16.9)	11.0 (6.6)	2.2 (2.4)	0.1 (0.3)	3.7 (1.1)
	66 × 52	3432	156	80.1 (20.9)	6.5 (4.5)	1.5 (2.1)	0.3 (0.9)	2.3 (2.2)
	66 × 51	3366	153	93.3 (15.7)	8.8 (5.8)	2.6 (2.1)	1.2 (1.8)	3.4 (1.3)
	66 × 50	3300	150	64.6 (13.6)	4.8(2.8)	1.1(0.9)	0.4(0.6)	0.8(0.9)

9 *vs* 9	66 × 47	3102	172	100.7 (11,9)	10.9 (5.1)	2.5 (1.5)	0.8 (0.8)	4.0 (1.2)
	66 x54	3564	198	96.3 (11.1)	10.8 (4.8)	2.8 (1.8)	0.7 (0.9)	4.7 (1.1)
	78 × 66	5148	286	106.0 (13.4)	15.5 (5.5)	4.9 (3.5)	1.3 (1.5)	4.0 (1.0)
	42 × 28	1176	65	85.3 (13.0)	5.9 (3.9)	0.6 (0.8)	0 (0)	5.0 (1.5)
	65 × 45	2925	162	97.5 (11.4)	10.8 (5.7)	3.3 (2.9)	1.1 (1.4)	4.1 (1.1)
	54 × 40	2160	120	87.4 (10.3)	8.6 (3.0)	2.6 (1.6)	0.8 (1.0)	4.1 (0.8)
	52 × 25	1300	72	83.1 (13.9)	4.6 (3.1)	1.1 (1.1)	0.3 (0.5)	4.7 (2.2)
	52 × 30	1560	87	83.7 (9.7)	6.5 (2.4)	1.2 (0.9)	0.1 (0.2)	4.1 (0.8)
	40 × 40	1600	89	96.8 (13.6)	8.0 (4.9)	1.2 (1.6)	0.2 (0.6)	4.5 (1.0)
	52 × 40	2080	116	87.7 (10.6)	8.4 (3.7)	1.7 (1.6)	0.4 (0.8)	4.5 (1.2)
	53 × 51	2703	150	113.3 (11.6)	14.7 (4.4)	3.6 (2.0)	0.4 (0.7)	4.0 (1.1)
	65 × 50	3250	181	60.6 (16.7)	3.7 (2.2)	0.6 (0.7)	0.3 (0.5)	0.9 (1.5)
	66 × 48	3168	176	94.3 (10.4)	11.0 (4.7)	4.0 (2.8)	1.3 (1.4)	4.2 (0.9)
	65 × 51	3315	184	104.6 (13.8)	13.5 (5.6)	4.3 (1.9)	1.4 (1.3)	4.1 (0.7)
	66 × 51	3366	187	103.5 (15.9)	13.9 (5.5)	3.9 (2.1)	1.0 (1.2)	4.5 (1.1)
	65 × 52	3380	188	64.2 (16.8)	4.3 (2.8)	1.1 (1.1)	0.9 (2.3)	0.7 (1.0)
	66 × 52	3432	191	101.4 (12.5)	12.7 (4.9)	3.7 (2.4)	1.1 (1.5)	4.5 (1.1)
	66 × 61	4026	223	101.0 (12.3)	12.7 (6.4)	4.0 (6.4)	1.4 (1.5)	4.0 (0.9)
	70 × 66	4620	257	115.1 (11.8)	17.0 (6.8)	4.8 (2.2)	1.4 (1.1)	4.3 (0.9)

9 *vs* 9 (+1f)	40 × 40	1600	84	104.6 (14.6)	12.5 (5.0)	2.7 (3.4)	0.3 (0.6)	5.8 (1.8)
	41 × 40	1640	86	104.3 (16.2)	9.8 (4.5)	0.4 (0.8)	0 (0)	3.6 (1.4)
	66 × 52	3432	181	67.3 (16.9)	4.0 (3.1)	1.0 (1.2)	0.2 (0.4)	1.3 (1.9)
	66 × 52	3432	181	95.5 (14.7)	9.8 (3.3)	2.0 (1.3)	0.4 (0.5)	3.6 (1.3)

8 *vs* 8	32 × 28	896	56	96.6 (12.9)	7.2 (4.2)	0.7 (1.0)	0 (0)	6.0 (1.6)
	40 × 35	1400	88	103.5 (14.0)	11.1 (4.0)	1.9 (1.2)	0.2 (0.3)	5.3 (1.1)
	40 × 40	1600	100	105.9 (9.9)	9.9 (4.0)	1.1 (1.6)	0.2 (0.5)	4.5 (1.1)
	45 × 40	1800	112.5	95.9 (15.1)	9.1 (4.6)	1.5 (1.3)	0.4 (0.9)	5.0 (1.2)
	52 × 35	1820	114	88.5 (9.7)	7.8 (3.6)	2.1 (1.2)	0.5 (0.5)	4.3 (0.9)
	52 × 40	2080	130	96.7 (10.5)	11.3 (3.7)	2.5 (1.5)	0.3 (0.4)	5.2 (1.0)
	53 × 40	2120	133	97.5 (12.2)	12.4 (4.6)	3.2 (1.7)	0.6 (0.6)	4.7 (1.0)
	50 × 40	2000	125	110.2 (13.1)	14.3 (5.4)	1.8 (1.0)	0.2 (0.5)	5.7 (1.0)
	53 × 45	2385	149	90.4 (8.3)	9.2 (1.9)	2.4 (1.0)	0.5 (0.7)	4.2 (0.7)
	55 × 52	2860	179	69.9 (17.0)	4.6 (2.0)	1.0 (1.1)	0.6 (1.0)	1.3 (1.4)
	70 × 46	3220	201	109.5 (10.6)	15.7 (4.8)	4.5 (1.5)	1.0 (0.9)	4.7 (0.7)
	66 × 52	3432	215	86.7 (8.9)	9.6 (4.8)	2.8 (1.9)	0.9 (1.5)	3.9 (1.1)
	66 × 50	3300	206	101.8 (11.0)	12.3 (4.3)	3.2 (1.8)	0.9 (1.1)	4.0 (0.6)

8 *vs* 8 (+1f)	40 × 36	1440	85	97.5 (13.8)	8.0 (4.3)	1.3 (1.5)	0 (0.1)	4.3 (1.3)
	54 × 36	1944	114	95.8 (10.3)	10.5 (4.3)	2.7 (1.4)	0.4 (0.5)	4.9 (1.3)
	55 × 45	2475	146	95.4 (12.5)	11.5 (4.8)	2.9 (2.2)	0.5 (0.6)	4.8 (1.2)
	52 × 28	1456	86	81.1 (12.4)	7.5 (3.8)	1.8 (1.3)	0.2 (0.5)	4.2 (1.4)
	50 × 25	1250	74	92.6 (9.9)	7.8 (1.9)	1.3 (0.9)	0.1 (0.2)	4.8 (0.8)
	52 × 25	1300	76	62.0 (18.2)	4.3 (2.9)	0.9 (0.9)	0.2 (0.4)	1.4 (1.9)
	40 × 40	1600	94	98.6 (9.1)	8.3 (4.0)	1.2 (1.5)	0.3 (0.7)	4.0 (1.2)
	41 × 40	1640	96	100.7 (11.7)	8.3 (3.5)	0.9 (1.1)	0.3 (0.7)	4.0 (0.6)
	47 × 40	1880	111	111.9 (12.6)	11.2 (2.9)	1.0 (0.9)	0 (0)	4.9 (0.7)
	53 × 47	2491	147	94.6 (8.1)	11.0 (3.3)	2.8 (1.8)	0.8 (0.7)	4.0 (1.0)
	66 × 45	2970	175	100.0 (11.7)	11.5 (3.2)	3.6 (2.1)	1.4 (1.5)	4.0 (1.0)
	65 × 51	3315	195	106.9 (21.8)	15.5 (6.4)	6.2 (4.0)	1.4 (1.2)	3.9 (1.7)
	52 × 40	2080	122	92.8 (12.8)	9.9 (4.4)	2.1 (1.6)	0.5 (1.2)	4.1 (1.1)

7 *vs* 7	53 × 40	2120	151	95.0 (9.9)	10.7 (3.2)	2.4 (1.1)	0.4 (0.4)	4.7 (0.8)
	40 × 40	1600	114	103.2 (15.7)	11.4 (5.1)	1.3 (1.2)	0.3 (0.7)	4.8 (1.3)

6 *vs* 6	40 × 36	1440	103	103.0 (13.1)	10.4 (4.4)	1.2 (1.1)	0.2 (0.7)	5.1 (1.1)
	47 × 40	1880	134	118.0 (17.4)	17.0 (8.4)	2.5 (2.8)	0.6 (1.4)	5.2 (1.1)
	52 × 25	1300	93	103.2 (11.2)	11.0 (3.7)	1.7 (1.3)	0.6 (0.9)	5.8 (1.3)
	52 × 22	1144	82	86.9 (12.6)	8.7 (4.2)	1.7 (1.4)	0.2 (0.4)	5.6 (1.4)
	40 × 35	1400	100	100.6 (13.8)	7.4 (5.0)	0.4 (0.6)	0 (0)	4.9 (1.5)
	50 × 30	1500	107	95.4 (5.9)	10.0 (3.0)	2.7 (2.0)	0.7 (0.9)	4.7 (0.9)
	52 × 40	2080	149	94.6 (9.3)	11.2 (3.9)	2.6 (1.6)	0.4 (0.6)	4.9 (1.0)
	52 × 47	2444	175	94.3 (12.0)	11.8 (4.1)	3.1 (2.1)	0.7 (1.3)	4.2 (0.9)

6 *vs* 6 (+1f)	42 × 30	1260	84	89.2 (9.3)	6.9 (2.9)	1.0 (0.9)	0.1 (0.3)	4.8 (1.3)
	40 × 36	1440	111	94.5 (11.8)	7.9 (3.6)	0.9 (0.9)	0.1 (0.3)	4.5 (1.4)

5 *vs* 5	40 × 36	1440	144	112.3(14.6)	11.7 (5.5)	1.9 (1.9)	0.2 (0.4)	5.9 (1.3)
	41 × 27	1107	111	100.1(10.1)	12.9 (4.8)	2.6 (1.8)	0.4 (0.7)	6.0 (1.2)
	40 × 30	1200	120	113.4(13.2)	15.1 (5.3)	3.5 (1.6)	0.4 (0.6)	6.0 (1.0)
	40 × 30	1200	120	106.1(5.2)	14.2 (3.2)	2.3 (1.7)	0.6 (0.8)	5.5 (1.3)
	40 × 20	800	80	95.2 (7.9)	8.9 (1.4)	2.3 (1.1)	0.3 (0.4)	4.7 (1.0)
	42 × 16	672	67	102.9(9.2)	12.9 (3.4)	2.7 (2.1)	0.4 (0.6)	6.0 (1.0)
	40 × 25	1000	100	103.8(10.6)	13.5 (4.6)	3.5 (2.9)	1.0 (1.8)	5.7 (1.1)
	35 × 30	1050	105	108.7(16.7)	14.3 (6.3)	1.4 (1.1)	0.1 (0.2)	6.9 (1.5)
	40 × 25	1000	100	70.1 (21.2)	7.1 (5.2)	1.4 (1.5)	0.1 (0.2)	2.5 (2.5)
	40 × 33	1320	132	123.9(9.2)	21.5 (4.9)	5.6 (1.8)	2.4 (1.9)	7.5 (1.8)
	40 × 35	1400	140	105.8(13.6)	13.1 (6.2)	1.8 (2.1)	0.2 (0.4)	6.0 (1.5)
	40 × 35	1400	140	123.4(15.6)	16.9 (5.7)	2.5 (1.4)	0.2 (0.4)	7.0 (1.0)
	40 × 38	1520	152	87.9 (7.7)	8.9 (3.9)	1.6 (0.7)	0 (0)	4.4 (1.1)
	40 × 40	1600	160	113.8(15.5)	13.0 (6.6)	2.2 (3.0)	0 (0.1)	6.1 (1.8)
	33 × 15	495	50	88.6 (8.5)	8.6 (4.1)	1.3 (1.3)	0 (0)	6.8 (1.2)
	33 × 33	1089	109	106.0(14.9)	11.4 (6.0)	1.0 (1.5)	0 (0.1)	6.3 (1.8)
	47 × 22	1034	103	112.1(10.9)	15.1 (6.0)	2.9 (1.9)	0.4 (0.6)	5.6 (1.4)
	42 × 28	1176	118	103.0(13.8)	11.4 (6.7)	1.6 (1.8)	0.2 (0.7)	5.6 (1.3)
	37 × 22	814	81.4	97.3 (9.2)	10.6 (3.8)	1.6 (1.0)	0.1 (0.4)	5.9 (1.3)
	37 × 33	1221	122	118.9(13.3)	15.5 (5.7)	2.0 (2.3)	0.2 (0.5)	7.3 (1.4)
	52 × 28	1456	146	109.6(14.5)	16.2 (6.8)	5.0 (3.3)	0.6 (1.0)	6.2 (1.5)

5 *vs* 5 (+1f)	43 × 35	1505	137	98.4 (11.8)	11.5 (3.9)	1.9 (1.0)	0.3 (0.3)	5.8 (1.0)
	45 × 35	1575	143	110.3(13.6)	16.5 (7.5)	3.8 (2.9)	0.5 (0.9)	5.8 (1.4)
	40 × 28	1120	102	84.3 (12.7)	6.9 (3.2)	0.8 (0.8)	0 (0.1)	3.1 (1.3)
	40 × 36	1440	131	108.1(20.2)	12.1 (6.0)	1.9 (2.1)	0.1 (0.4)	4.7 (1.8)
	40 × 25	1000	91	92.2 (11.3)	7.3 (3.9)	0.5 (0.4)	0.1 (0.2)	4.9 (1.1)
	25 × 22	550	50	89.0 (12.2)	3.2 (1.7)	0.2 (0.4)	0 (0)	4.3 (1.0)
	40 × 26	1040	95	98.8 (10.0)	13.4 (3.9)	3.2 (2.0)	0.4 (0.5)	5.4 (1.2)
	50 × 25	1250	114	63.4 (9.1)	5.7 (3.0)	1.4 (1.2)	0.3 (0.5)	0.7 (0.3)
	45 × 30	1350	123	94.9 (8.2)	6.5 (2.0)	1.1 (1.0)	0.1 (0.3)	3.6 (1.3)
	52 × 36	1008	92	106.1(13.3)	14.1 (5.2)	3.4 (1.6)	0.7 (0.6)	4.4 (0.8)
	40 × 27	1080	98	85.9 (9.5)	7.3 (2.9)	0.9 (1.0)	0.1 (0.4)	4.4 (1.1)
	50 × 20	1000	91	58.7 (16.0)	5.2 (2.4)	2.1 (2.1)	0.7 (1.4)	1.3 (1.9)
	43 × 28	1204	109	96.7 (10.4)	9.5 (2.4)	2.1 (0.9)	0.4 (0.7)	4.5 (0.7)
	40 × 40	1600	145	93.8 (11.9)	10.9 (3.3)	2.1 (1.6)	0.1 (0.1)	4.9 (1.0)
	50 × 34	1700	155	110.1(13.2)	15.9 (6.1)	3.0 (2.0)	0.5 (0.9)	5.9 (1.2)
	50 × 40	2000	182	109.6(13.5)	15.6 (5.6)	4.7 (2.6)	1.1 (1.7)	5.1 (1.3)
	52 × 40	2080	160	97.0 (9.1)	10.7 (3.6)	3.2 (2.2)	0.7 (0.9)	4.4 (1.1)

4 vs 4	30 × 20	600	754	86.5 (9.0)	6.3 (2.0)	0.4 (0.3)	0(0)	6.1 (1.6)
	40 × 25	1000	125	108.2(11.1)	16.4 (4.2)	1.8 (1.9)	0 (0.1)	6.8 (1.0)
	40 × 30	1200	150	111.5(14.5)	18.9 (6.4)	3.5 (2.6)	0.1 (0.3)	6.2 (1.4)
	40 × 35	1400	175	128.2(12.8)	17.8 (6.5)	4.2 (2.2)	0.2 (0.5)	6.7 (1.4)
	32 × 28	896	112	116.4(15.9)	13.8 (6.6)	1.0 (1.5)	0 (0.0)	7.2 (1.8)
	40 × 25	1000	125	111.8(12.5)	13.8 (3.1)	2.0 (1.2)	0.1 (0.3)	6.4 (1.1)
	40 × 35	1400	175	100.4(9.8)	8.8 (2.9)	0.5 (0.6)	0(0)	6.3 (1.9)

4 vs 4 (+1f)	36 × 22	792	88	99.9 (11.2)	13.8 (4.4)	2.1 (1.2)	0.1 (0.1)	6.2 (0.9)
	43 × 25	1075	119	102.9(8.6)	15.2 (5.2)	3.4 (2.2)	0.4 (0.7)	6.2 (1.7)
	40 × 20	800	89	114.1(11.8)	14.4 (11.8)	2.6 (1.7)	0.3 (0.7)	7.2 (1.3)
	30 × 30	900	100	110.6(12.0)	9.3 (5.5)	0.9 (1.1)	0.1 (0.2)	7.2 (1.5)
	40 × 20	800	89	97.7 (6.8)	13.6 (5.0)	3.9 (1.5)	0.4 (0.5)	3.8 (1.1)
	40 × 40	1600	178	119.0(22.8)	20.4 (12.9)	3.7 (4.4)	0.8 (0.8)	5.7 (2.6)
	40 × 23	920	102	90.0 (8.6)	7.6 (2.3)	0.9 (0.9)	0 (0)	5.1 (0.9)
	43 × 22	946	105	99.0 (12.8)	12.0 (4.9)	2.7 (1.7)	0.3 (0.7)	4.8 (0.8)
	47 × 40	1880	209	134.4(19.9)	23.4 (8.4)	4.5 (2.5)	0.2 (0.6)	6.7 (1.6)
	40 × 25	1000	111	104.3(9.5)	12.8 (3.2)	2.7 (1.8)	0.4 (0.8)	6.0 (1.4)
	34 × 30	1020	113	108.4(6.5)	13.5 (4.0)	2.6 (2.1)	0.3 (0.8)	6.6 (1.2)
	40 × 31	1240	138	116.3(10.5)	16.2 (4.7)	2.7 (1.4)	0.1 (0.2)	6.4 (1.0)

3 vs 3	40 × 32	1280	213	88.9 (9.6)	7.6 (3.8)	1.5 (1.2)	0 (0.1)	6.4 (1.4)
	30 × 20	600	100	94.9 (8.1)	7.8 (2.4)	0.6 (0.5)	0.1 (0.3)	6.8 (1.5)
	32 × 16	512	85	112.3(11.4)	17.2 (5.1)	2.7 (2.5)	0.4 (1.3)	8.7 (1.5)

3 vs 3 (+1f)	25 × 22	550	79	102.8(16.1)	9.1 (6.5)	1.5 (2.1)	0 (0)	6.0 (1.9)
	32 × 16	512	73	105.7(7.9)	14.8 (4.1)	2.3 (1.3)	0.3 (0.5)	7.0 (1.0)
	37 × 20	740	106	118.3(13.0)	22.4 (5.3)	5.9 (5.2)	0.9 (1.5)	6.3 (1.9)

10 vs 9	70 × 65	4550	239	109.3(11.5)	14.2 (5.1)	5.3 (2.6)	2.4 (1.4)	4.7 (0.8)
	54 × 54	2916	153	93.4 (10.6)	8.9 (4.0)	2.5 (2.0)	0.6 (0.7)	4.6 (0.9)
	66 × 35	2310	122	103.8(11.8)	11.8 (5.7)	2.7 (1.8)	0.7 (0.9)	5.3 (1.3)

6 vs 3 (+1f)	37 × 20	740	74	103.2(8.2)	15.1 (4.1)	5.0 (2.6)	1.5 (1.2)	5.9 (1.3)

7 vs 6	53 × 27	1431	110	88.7 (8.3)	10.6 (3.7)	2.0 (1.7)	0.5 (0.7)	3.7 (1.0)

The small-sided games are split for the number of players and pitch size (width × length). The total pitch area (m^2^) and area per player (m^2^ · player) have been calculated. The number of floaters, when required, is reported between bracket (i.e., one floater: +1f, two floaters: +2f, etc.). Average locomotor demands are reported for total distance (TD), high-speed running (HSR), very high-speed running (VHSR), sprint (SPR) and acceleration/Deceleration (Acc/Dec). Data are reported as mean (SD).

**Supplementary table 4:** Pitch size and locomotor demands during small-sided games in U18 soccer players.

N° Player	m	m^2^	m^2^⋅player	TD	HSR	VHSR	SPR	Acc/Dec
10 *vs* 10	60 × 52	3120	156	102.8 (8.4)	10.3 (4.6)	2.3 (2.0)	0.4 (0.7)	4.5 (0.9)

9 *vs* 9	50 × 60	3000	166.7	88.8 (6.8)	9.2 (2.8)	1.9 (0.9)	0.5 (0.6)	3.0 (0.8)
	40 × 52	2080	115.6	88.1 (18.7)	8.2 (5.2)	2.2 (2.1)	0.5 (0.8)	3.3 (1.6)

8 *vs* 8	65 × 52	3380	211.3	99.9 (7.5)	10.9 (2.6)	2.3 (1.2)	0.4 (0.3)	4.5 (0.8)
	50 × 60	3000	187.5	104.8 (9.6)	12.1 (4.7)	3.0 (1.8)	0.5 (0.6)	5.0 (0.8)
	53 × 40	2120	132.5	58.9 (3.8)	4.4 (1.8)	0.7 (0.6)	0.1 (0.3)	4.0 (1.1)
	35 × 60	2100	131.3	108.2 (8.7)	12.0 (4.3)	3.2 (1.6)	0.5 (0.6)	4.3 (0.7)
	40 × 52	2080	130.0	92.3 (8.2)	9.4 (3.4)	2.5 (1.2)	0.5 (0.4)	4.4 (0.7)
	40 × 50	2000	125.0	96.0 (9.4)	10.2 (2.8)	1.8 (1.2)	0.4 (0.5)	4.6 (0.9)
	40 × 40	1600	100.0	84.6 (8.2)	7.2 (2.6)	1.6 (1.3)	0.2 (0.3)	4.7 (1.0)

8 *vs* 8 (+1f)	60 × 50	3000	176.5	92.1 (10.0)	10.2 (3.8)	2.6 (1.0)	0.4 (0.5)	2.7 (0.5)
	40 × 60	2400	141.2	94.7 (7.7)	10.9 (2.3)	3.9 (1.7)	1.7 (1.5)	3.3 (0.5)
	40 × 40	1600	94.1	83.5 (10.5)	6.7 (3.0)	1.1 (0.8)	0.1 (0.2)	4.5 (1.0)
	35 × 40	1400	82.4	90.5 (9.0)	6.3 (3.1)	1.2 (1.1)	0.2 (0.5)	4.5 (1.0)

7 vs 7	48 × 51	2448	174.9	99.9 (6.7)	10.8 (4.1)	2.1 (1.0)	0.3 (0.5)	4.0 (0.8)
	53 × 40	2120	151.4	100.8 (3.0)	9.3 (2.7)	2.4 (1.1)	0.5 (0.4)	5.6 (0.9)
	40 × 52	2080	148.6	97.0 (13.2)	9.7 (4.6)	2.3 (1.4)	0.3 (0.3)	4.2 (1.3)
	40 × 50	2000	142.9	94.4 (7.2)	10.3 (4.5)	2.1 (1.9)	0.4 (0.5)	3.9 (0.8)
	38 × 52	1976	141.1	104.2 (9.2)	12.6 (3.7)	2.8 (1.4)	0.5 (0.7)	5.1 (0.8)
	35 × 45	1575	112.5	93.2 (5.8)	7.1 (0.6)	1.3 (0.8)	0.5 (0.7)	3.7 (0.5)
	40 × 25	1000	71.4	85.8 (8.3)	5.5 (2.9)	0.5 (0.5)	0.1 (0.2)	4.1 (0.9)

8 *vs* 6	40 × 52	2080	148.6	99.0 (7.8)	9.5 (3.0)	1.9 (1.0)	0.2 (0.3)	4.2 (0.7)

6 vs 6	34 × 50	1700	141.7	84.0 (9.4)	9.4 (3.4)	3.2 (2.6)	1.4 (1.5)	3.9 (1.0)
	40 × 40	1600	133.3	93.4 (8.4)	8.1 (2.4)	1.2 (0.9)	0.2 (0.5)	4.9 (0.7)
	40 × 38	1520	126.7	97.3 (6.9)	8.9 (1.8)	1.7 (0.7)	0.4 (0.5)	4.5 (0.6)
	35 × 40	1400	116.7	91.2 (12.6)	7.6 (4.8)	1.1 (1.2)	0.1 (0.3)	4.8 (1.1)
	30 × 35	1050	87.5	82.4 (6.3)	6.0 (2.5)	0.6 (0.6)	0.0 (0.0)	4.6 (1.1)

6 *vs* 6 (+1f)	46 × 48	2208	169.8	106.6 (9.6)	12.4 (4.0)	2.5 (1.8)	0.9 (1.3)	4.5 (0.9)
	34 × 60	2040	156.9	80.7 (9.8)	8.4 (3.2)	1.9 (1.2)	0.4 (0.3)	3.9 (0.5)
	40 × 40	1600	123.1	91.2 (6.4)	7.3 (3.1)	1.1 (1.4)	0.1 (0.2)	5.0 (1.2)

5 vs 5	44 × 34	1496	149,6	83.8 (9.9)	5.9 (3.1)	1.0 (1.2)	0.4 (0.5)	4.0 (1.4)
	40 × 37	1480	148.0	89.7 (5.7)	8.0 (3.1)	1.6 (1.2)	0.2 (0.3)	4.5 (0.6)
	30 × 34	1020	102.0	94.6 (5.6)	7.6 (2.6)	1.5 (1.2)	0.2 (0.2)	5.1 (1.0)
	25 × 38	950	95.0	97.6 (7.4)	9.6 (2.8)	1.2 (0.9)	0.3 (0.4)	5.7 (1.1)
	30 × 30	900	90.0	84.6 (6.4)	6.9 (2.7)	0.7 (0.4)	0.0 (0.1)	4.4 (0.7)

4 *vs* 4	40 × 35	1400	175.0	89.1 (11.6)	6.0 (3.7)	0.8 (1.1)	0.1 (0.3)	5.5 (1.9)
	30 × 40	1200	150.0	82.3 (20.3)	9.1 (3.7)	1.6 (1.3)	0.1 (0.3)	4.4 (1.0)

4 *vs* 4 (+1f)	30 × 35	1050	116.7	110.5 (9.0)	10.6 (4.2)	1.4 (1.8)	0.0 (0.2)	5.9 (1.5)
	30 × 30	900	100.0	101.4 (6.4)	8.2 (3.0)	0.8 (0.8)	0.0 (0.0)	6.6 (1.1)

3 *vs* 3 (+1f)	25 × 30	750	107,1	128.7 (13.4)	20.8 (6.7)	1.5 (2.0)	0.1 (0.2)	9.9 (1.2)
	15 × 25	375	53.6	81.0 (7.7)	4.0 (2.6)	0.5 (0.6)	0.0 (0.0)	5.0 (1.3)

3 *vs* 3	20 × 25	500	83.3	102.2 (8.0)	14.3 (4.2)	2.0 (1.7)	0.5 (0.8)	8.2 (1.5)

2 *vs* 2	20 × 30	600	150	138.3 (9.2)	28.6 (7.5)	6.3 (4.0)	0.3 (0.8)	14.3 (2.5)

The small-sided games are split for the number of players and pitch size (width × length). The total pitch area (m^2^) and area per player (m^2^ · player) have been calculated. The number of floaters, when required, is reported between bracket (i.e., one floater: +1f; two floaters: +2f). The total pitch area (m^2^) and area per player (m^2^ · player) have been calculated. Average locomotor demands are reported for total distance (TD), high-speed running (HSR), very high-speed running (VHSR), sprint (SPR) and acceleration/Deceleration (Acc/Dec). Data are reported as mean (SD).

**Supplementary table 5:** Pitch size and locomotor demands during small-sided games in U19 soccer players.

N° Players	m	m^2^	m^2^⋅player	TD	HSR	VHSR	SPR	Acc/Dec
10 *vs* 10	66 × 70	4620	231.0	109.3 (15.4)	13.3 (6.7)	4.8 (3.4)	2.1 (2.2)	3.5 (1.4)
	65 × 75	4875	243.8	116.7 (12.1)	17.0 (4.6)	4.8 (3.3)	1.5 (1.8)	4.4 (1.1)
	66 × 90	5940	297.0	116.4 (15.1)	14.9 (5.5)	5.8 (3.1)	3.1 (2.4)	3.2 (1.2)
	65 × 90	5850	292.5	129.2 (11.5)	20.7 (3.3)	8.7 (3.5)	3.8 (2.2)	4.1 (1.2)
	33 × 32	1056.24	52.8	84.3 (9.1)	2.8 (2.0)	0.4 (0.6)	0.0 (0.1)	4.0 (1.0)
	40 × 40	1600	80.0	96.5 (10.9)	5.9 (2.6)	1.2 (1.2)	0.2 (0.4)	4.0 (1.1)
	50 × 35	1750	87.5	96.6 (10.6)	5.6 (2.3)	0.8 (0.8)	0.1 (0.2)	4.1 (0.8)
	43 × 43	1806	90.3	99.3 (9.9)	6.4 (2.7)	1.5 (1.7)	0.9 (1.5)	4.2 (1.3)
	50 × 36	1800	90.0	101.0 (16.0)	6.7 (3.1)	1.0 (1.0)	0.2 (0.4)	4.5 (1.1)
	50 × 40	2000	100.0	98.3 (9.3)	6.3 (2.5)	1.1 (0.7)	0.1 (0.2)	4.7 (0.8)
	42 × 48	2016	100.8	97.7 (11.0)	7.0 (3.1)	1.6 (1.3)	0.4 (0.7)	4.2 (1.1)
	40 × 52	2080	104.0	92.3 (13.3)	8.0 (2.7)	1.4 (0.8)	0.2 (0.3)	3.4 (0.6)
	40.9 × 51.1	2090	104.5	97.1 (9.5)	8.0 (2.9)	1.9 (1.3)	0.4 (0.7)	4.1 (1.1)
	45.8 × 50.2	2300	115.0	100.1 (9.8)	8.9 (2.7)	2.3 (1.6)	0.8 (1.0)	4.1 (1.2)
	50 × 45	2250	112.5	96.7 (10.9)	5.7 (2.3)	0.9 (0.8)	0.0 (0.1)	4.2 (1.0)
	40 × 60	2400	120.0	100.0 (8.5)	8.2 (3.2)	2.3 (1.8)	0.8 (0.9)	4.1 (1.2)
	50 × 52	2600	130.0	99.7 (10.7)	8.8 (3.1)	2.1 (1.3)	0.4 (0.6)	4.2 (1.4)
	52 × 52	2704	135.2	99.2 (7.9)	8.1 (2.5)	2.3 (1.8)	0.7 (0.7)	3.8 (0.9)
	50 × 55	2750	137.5	101.4 (7.7)	9.7 (2.7)	2.7 (1.7)	0.9 (1.3)	4.6 (0.8)
	50 × 60	3000	150.0	97.5 (11.0)	9.3 (3.9)	2.8 (1.7)	0.9 (1.1)	4.2 (1.1)
	50 × 61	3050	152.5	105.9 (9.4)	11.1 (3.0)	3.3 (1.5)	1.2 (1.2)	3.4 (0.9)
	52 × 65	3380	169.0	101.2 (13.6)	8.8 (4.4)	2.4 (2.2)	0.9 (1.6)	3.9 (1.2)
	50 × 70	3500	175.0	104.6 (12.5)	10.2 (4.5)	3.3 (2.2)	1.3 (1.3)	4.0 (0.9)
	60 × 65	3900	195.0	113.6 (11.7)	12.2 (4.1)	4.2 (2.3)	1.7 (1.8)	4.0 (0.7)
	50 × 80	4000	200.0	84.5 (8.4)	8.4 (2.7)	2.3 (1.1)	0.7 (0.8)	3.7 (0.8)
	55 × 75	4125	206.3	111.7 (11.5)	13.5 (4.5)	3.6 (1.9)	1.2 (1.1)	4.0 (1.0)
	65 × 70	4550	227.5	109.0 (11.7)	13.1 (4.1)	4.3 (2.6)	2.0 (2.0)	4.0 (0.8)
	60 × 80	4800	240.0	94.7 (10.7)	9.9 (3.1)	2.9 (1.6)	1.4 (1.2)	3.0 (0.8)
	65.5 × 104	6812	340.6	122.8 (12.5)	18.3 (5.1)	6.1 (2.6)	3.1 (2.1)	3.4 (0.8)
	66 × 104	6864	343.2	135.0 (11.7)	22.8 (7.3)	8.7 (3.8)	4.8 (3.9)	3.6 (1.3)

10 *vs* 10 (+1f)	66 × 104	6864	326.9	135.0 (13.5)	21.8 (7.5)	8.3 (4.0)	4.9 (3.2)	3.6 (1.0)
	66 × 90	5940	282.9	126.4 (10.7)	18.4 (4.4)	6.4 (2.3)	3.4 (3.3)	3.9 (1.3)
	66 × 70	4620	220.0	124.6 (10.2)	19.0 (3.3)	7.1 (3.6)	1.7 (1.6)	3.9 (1.3)
	40 × 60	2400	114.3	98.4 (9.0)	7.2 (2.7)	2.1 (1.8)	0.3 (0.4)	4.0 (1.0)
	30 × 35	1050	50.0	95.4 (9.4)	4.8 (2.6)	0.7 (0.8)	0.2 (0.3)	4.1 (1.1)
	35 × 50	1750	83.3	101.4 (7.4)	8.0 (2.8)	2.6 (1.7)	1.0 (1.0)	4.5 (1.2)
	35 × 54	1890	90.0	97.8 (8.0)	8.9 (2.3)	2.1 (1.4)	0.5 (0.6)	4.2 (0.8)
	40 × 55	2200	104.8	99.1 (7.9)	8.4 (2.7)	2.1 (1.1)	0.5 (0.5)	4.1 (0.8)
	35 × 40	1400	66.7	90.9 (8.9)	4.6 (2.2)	0.8 (0.8)	0.2 (0.3)	4.0 (1.2)
	50 × 35	1750	83.3	93.3 (14.1)	5.2 (1.9)	0.5 (0.4)	0.1 (0.1)	3.8 (0.8)
	45 × 40	1800	85.7	101.4 (12.4)	5.6 (1.9)	0.9 (0.6)	0.1 (0.1)	4.1 (0.7)
	50 × 40	2000	95.2	96.8 (9.2)	5.9 (2.3)	1.0 (0.9)	0.1 (0.1)	4.2 (1.0)
	40 × 50	2000	95.2	98.9 (10.1)	7.2 (3.7)	2.1 (1.6)	1.0 (1.3)	3.9 (1.2)
	45 × 45	2025	96.4	96.5 (9.7)	7.1 (2.8)	1.2 (0.8)	0.4 (0.8)	4.7 (1.1)
	40 × 52	2080	99.0	101.7 (8.9)	8.1 (3.0)	1.9 (1.3)	0.5 (0.6)	3.9 (1.1)
	45 × 50	2250	107.1	97.2 (10.3)	9.2 (3.0)	3.3 (2.2)	1.3 (2.1)	4.7 (1.3)
	50 × 50	2500	119.0	103.8 (8.8)	9.6 (2.8)	2.7 (1.7)	0.3 (0.3)	4.6 (1.0)
	50 × 52	2600	123.8	103.3 (10.2)	9.6 (3.6)	2.4 (1.8)	1.0 (1.4)	4.2 (1.1)
	50 × 70	3500	166.7	109.6 (12.9)	10.5 (3.4)	3.0 (2.2)	1.5 (1.8)	3.7 (1.0)
	50 × 80	4000	190.5	114.4 (8.8)	12.1 (4.6)	3.9 (2.7)	1.4 (1.4)	3.7 (1.0)

10 *vs* 10 (+2f)	40 × 52	2080	94.5	102.6 (11.0)	8.3 (3.1)	1.9 (1.5)	0.5 (0.5)	3.8 (0.6)
	40 × 55	2200	100.0	99.7 (8.9)	8.6 (4.4)	2.2 (1.5)	0.6 (0.9)	3.6 (1.1)
	65 × 80	5200	236.4	109.3 (7.5)	11.9 (3.6)	3.3 (2.1)	1.0 (1.2)	3.4 (0.9)

9 *vs* 9	40 × 40	1600	88.9	95.6 (14.3)	6.0 (2.4)	1.2 (1.0)	0.3 (0.5)	4.5 (1.3)
	40 × 55	2200	122.2	97.6 (7.6)	7.3 (2.9)	1.8 (1.5)	0.2 (0.4)	3.4 (1.2)
	40 × 50	2000	111.1	100.9 (10.6)	7.0 (3.4)	1.8 (1.6)	0.5 (1.0)	3.6 (1.2)
	40.8 × 51.2	2089	116.1	101.0 (9.5)	8.9 (3.0)	2.1 (1.5)	0.6 (0.8)	4.2 (1.0)
	40 × 54	2160	120.0	96.5 (7.0)	6.1 (2.3)	1.6 (1.2)	0.6 (0.7)	3.1 (0.9)
	50 × 52	2600	144.4	85.6 (14.0)	6.1 (2.7)	1.1 (0.4)	0.2 (0.1)	3.5 (1.1)
	65 × 70	4550	252.8	107.3 (10.2)	13.3 (4.5)	4.5 (2.2)	1.3 (1.2)	3.6 (0.8)
	67 × 70	4690	260.6	124.8 (7.9)	14.0 (4.8)	4.2 (2.1)	1.4 (1.6)	4.3 (0.8)
	66 × 104	6864	381.3	125.0 (6.4)	17.7 (4.0)	5.7 (1.9)	2.1 (1.1)	3.3 (0.6)

9 *vs* 9 (+1f)	68 × 104	7072	372.2	138.0 (11.4)	24.2 (6.5)	10.6 (3.1)	6.3 (3.1)	3.7 (0.9)
	32 × 38	1216	64.0	95.1 (7.3)	5.6 (1.9)	1.3 (0.9)	0.3 (0.5)	4.3 (1.1)
	40 × 45	1800	94.7	95.5 (7.9)	7.2 (3.1)	2.1 (1.1)	0.8 (0.8)	3.8 (1.1)
	35 × 35	1225	64.5	96.6 (7.1)	6.2 (2.1)	0.9 (0.8)	0.1 (0.2)	4.7 (1.3)
	40 × 40	1600	84.2	96.6 (9.4)	6.2 (2.8)	1.1 (1.1)	0.2 (0.3)	4.3 (1.0)
	40 × 52	2080	109.5	100.7 (10.0)	8.4 (3.6)	2.0 (1.2)	0.6 (0.6)	4.0 (1.1)
	45 × 50	2250	118.4	102.1 (13.9)	10.6 (4.7)	2.0 (1.3)	0.2 (0.2)	4.7 (1.0)

9 *vs* 9 (+2f)	30 × 35	1050	52.5	83.4 (8.7)	2.8 (2.0)	0.2 (0.4)	0.0 (0.1)	3.4 (1.0)
	35 × 30	1050	52.5	86.4 (8.0)	2.5 (2.3)	0.3 (0.6)	0.1 (0.3)	3.1 (1.1)
	40 × 30	1200	60.0	89.5 (8.1)	3.1 (2.1)	0.3 (0.4)	0.0 (0.0)	3.5 (1.1)

8 *vs* 8 (+2f)	30 × 30	900	50.0	85.6 (9.0)	2.7 (1.7)	0.4 (0.6)	0.0 (0.0)	4.7 (1.5)
	35 × 35	1225	68.1	93.3 (10.5)	6.8 (2.9)	1.7 (1.6)	0.2 (0.3)	4.8 (1.2)

8 *vs* 7	50 × 60	3000	200.0	86.4 (7.4)	9.1 (3.2)	2.1 (1.0)	0.4 (0.8)	3.4 (0.7)
7 *vs* 7	28 × 30	840	60	96.7 (6.3)	5.8 (2.5)	1.1 (0.8)	0.1 (0.2)	4.4 (1.1)
	33 × 52	1716	122.6	98.6 (8.0)	8.9 (3.1)	2.2 (1.7)	0.6 (0.8)	4.8 (1.2)
	32 × 40	1280	91.4	95.9 (8.8)	5.9 (2.0)	0.8 (0.8)	0.0 (0.0)	4.9 (1.6)
	40 × 48	1920	137.1	104.4 (6.7)	8.9 (2.5)	2.4 (1.5)	0.7 (1.1)	4.9 (1.3)
	25 × 25	625	44.6	84.8 (10.7)	2.7 (1.6)	0.2 (0.2)	0.0 (0.0)	4.6 (0.9)
	25 × 35	875	62.5	95.0 (8.2)	6.1 (2.8)	0.9 (0.7)	0.0 (0.0)	5.0 (0.7)
	30 × 40	1200	85.7	103.1 (8.7)	8.4 (3.6)	1.6 (1.3)	0.1 (0.2)	4.9 (1.1)
	35 × 40	1400	100.0	90.3 (7.3)	8.2 (1.6)	1.8 (0.8)	0.2 (0.2)	4.3 (0.5)
	35 × 45	1575	112.5	99.5 (11.2)	7.8 (3.1)	1.9 (1.1)	0.4 (0.4)	4.1 (1.2)
	40 × 45	1800	128.6	99.8 (7.4)	8.7 (3.1)	2.0 (1.1)	0.3 (0.3)	4.6 (1.1)
	40 × 52	2080	148.6	114.8 (19.6)	13.4 (6.6)	3.1 (2.2)	0.8 (0.9)	4.7 (1.0)
	45 × 70	3150	225.0	106.2 (7.3)	14.2 (4.0)	5.2 (3.7)	2.3 (1.9)	3.3 (0.8)
	50 × 70	3500	250.0	118.6 (12.1)	16.6 (5.1)	5.6 (2.3)	2.4 (2.0)	3.9 (0.9)

7 *vs* 7 (+1f)	25 × 30	750	50.0	82.9 (7.8)	3.0 (2.0)	0.2 (0.4)	0.0 (0.1)	4.0 (1.0)
	30 × 30	900	60.0	88.6 (9.2)	3.8 (1.6)	0.3 (0.3)	0.0 (0.1)	4.2 (1.2)
	30 × 40	1200	80.0	94.0 (8.8)	7.9 (3.9)	1.7 (1.3)	0.3 (0.5)	4.9 (1.1)
	40 × 52	2080	138.7	98.8 (12.7)	9.2 (4.4)	2.0 (1.6)	0.2 (0.5)	3.3 (0.8)
	50 × 50	2500	166.7	96.3 (14.1)	7.9 (4.7)	1.7 (1.7)	0.5 (0.8)	3.6 (1.3)
	50 × 70	3500	233.3	114.1 (14.7)	12.7 (6.1)	5.4 (3.3)	2.5 (1.6)	3.7 (0.8)
	50 × 75	3750	250.0	119.6 (14.0)	15.4 (7.5)	5.1 (2.2)	2.8 (2.7)	3.2 (1.0)

7 *vs* 7 (+2f)	25 × 30	750	46.9	89.6 (11.3)	2.7 (1.6)	0.3 (0.5)	0.0 (0.1)	3.9 (1.2)
6 *vs* 6	35 × 45	1575	131.3	93.0 (9.1)	8.9 (3.6)	1.8 (1.2)	0.3 (0.6)	3.9 (1.5)
	18 × 25	450	37.5	94.6 (8.6)	4.9 (1.8)	0.4 (0.3)	0.0 (0.1)	3.8 (0.8)
	25 × 20	500	41.7	92.4 (10.9)	4.7 (2.2)	0.4 (0.5)	0.0 (0.0)	4.5 (0.9)
	30 × 32	960	80.0	94.3 (10.1)	6.2 (3.0)	1.1 (0.8)	0.3 (0.4)	4.6 (1.5)
	20 × 35	700	58.3	88.2 (7.7)	4.9 (1.9)	0.7 (0.6)	0.1 (0.2)	5.3 (1.2)
	30 × 30	900	75.0	99.6 (8.1)	6.5 (2.6)	1.0 (0.8)	0.2 (0.3)	5.0 (1.1)
	30 × 32	960	80.0	95.1 (10.9)	6.6 (3.6)	0.8 (1.1)	0.2 (0.4)	4.9 (1.3)
	30 × 35	1050	87.5	99.4 (7.6)	6.7 (3.2)	1.5 (1.0)	0.1 (0.1)	3.9 (0.8)
	30 × 40	1200	100.0	96.2 (8.0)	8.2 (2.6)	1.7 (1.0)	0.3 (0.4)	4.9 (1.2)
	35 × 35	1225	102.1	101.3 (13.7)	8.6 (3.9)	1.5 (1.2)	0.2 (0.4)	5.4 (1.4)
	35 × 40	1400	116.7	100.6 (9.4)	8.5 (2.5)	1.8 (1.1)	0.3 (0.5)	4.1 (1.0)
	36 × 42	1512	126.0	102.0 (7.9)	8.3 (3.7)	1.8 (1.7)	0.3 (0.4)	3.9 (0.9)
	50 × 60	3000	250.0	122.5 (10.0)	15.1 (3.4)	3.7 (2.5)	0.9 (1.1)	4.1 (1.5)

6 *vs* 6 (+1f)	20 × 30	600	46.2	80.3 (13.5)	2.5 (2.0)	0.1 (0.3)	0.0 (0.1)	4.0 (1.4)
	35 × 40	1400	107.7	90.0 (11.5)	6.7 (3.3)	1.6 (1.2)	0.1 (0.3)	3.9 (1.1)
	40 × 35	1400	107.7	104.3 (2.7)	9.6 (1.8)	1.8 (0.7)	0.3 (0.3)	4.9 (1.2)
	34 × 42	1428	109.8	106.8 (9.8)	10.5 (3.7)	2.4 (0.9)	0.5 (0.7)	4.8 (1.2)

6 *vs* 6 (+2f)	30 × 30	900	64.3	95.5 (17.7)	5.9 (4.0)	0.5 (0.5)	0.0 (0.0)	3.4 (1.3)

5 *vs* 5	20 × 25	500	50.0	93.4 (6.9)	5.5 (1.8)	0.4 (0.4)	0.0 (0.1)	5.4 (1.0)
	50 × 52	2600	260.0	128.9 (13.0)	22.6 (7.1)	7.2 (2.5)	1.6 (1.6)	6.0 (1.6)
	30 × 40	1200	120.0	104.2 (7.9)	12.6 (2.5)	2.3 (1.4)	0.4 (0.5)	4.9 (1.5)
	20 × 20	400	40.0	111.8 (6.2)	7.0 (2.8)	0.3 (0.4)	0.0 (0.0)	5.3 (0.8)
	32 × 32	1024	102.4	96.8 (6.2)	6.8 (1.8)	0.9 (0.6)	0.1 (0.2)	4.9 (0.9)
	28 × 32	896	89.6	111.1 (12.9)	11.4 (5.3)	2.0 (1.1)	0.2 (0.5)	6.2 (1.1)
	25 × 22	550	55.0	101.7 (3.8)	5.6 (2.8)	0.4 (0.4)	0.0 (0.0)	4.8 (0.7)
	20 × 30	600	60.0	91.3 (8.5)	4.4 (2.2)	0.3 (0.4)	0.0 (0.0)	4.7 (1.2)
	32 × 30	960	96.0	106.2 (8.1)	10.3 (2.4)	1.8 (1.5)	0.2 (0.3)	5.8 (1.2)
	25 × 30	750	75.0	102.5 (7.7)	9.8 (2.7)	1.1 (0.7)	0.0 (0.0)	5.7 (0.8)
	30 × 30	900	90.0	106.1 (7.8)	9.0 (2.6)	1.5 (1.3)	0.1 (0.3)	6.1 (1.3)
	30 × 32	960	96.0	107.3 (10.5)	9.8 (3.9)	1.5 (1.1)	0.2 (0.3)	6.0 (1.4)
	34 × 37	1258	125.8	104.8 (10.4)	10.8 (4.1)	2.5 (1.3)	0.8 (0.6)	5.3 (0.9)
	50 × 35	1750	175.0	123.9 (10.5)	17.0 (4.1)	5.1 (2.2)	0.7 (0.9)	5.3 (1.2)
	40 × 50	2000	200.0	110.0 (9.8)	14.9 (3.1)	4.0 (1.7)	1.3 (1.2)	4.9 (1.1)
	40 × 52	2080	208.0	116.7 (14.9)	15.2 (5.2)	4.4 (2.3)	1.5 (1.7)	5.0 (1.3)

5 *vs* 5 (+1f)	25 × 20	500	45.5	86.2 (9.1)	3.0 (1.4)	0.5 (0.7)	0.0 (0.1)	5.1 (1.3)
	35 × 30	1050	95.5	99.7 (11.8)	8.2 (3.2)	1.2 (0.9)	0.1 (0.2)	4.8 (1.5)
	40 × 50	2000	181.8	130.0 (11.0)	19.8 (4.8)	6.2 (2.7)	0.9 (0.8)	5.2 (1.2)
	40 × 52	2080	189.1	126.4 (15.4)	18.5 (7.0)	5.2 (2.6)	0.8 (1.1)	4.9 (1.1)

4 *vs* 4	20 × 20	400	50.0	97.2 (8.0)	4.7 (2.1)	0.2 (0.2)	0.0 (0.0)	5.8 (1.3)
	28 × 32	896	112.0	105.8 (6.6)	10.9 (3.9)	1.4 (1.0)	0.1 (0.2)	5.9 (1.3)
	30 × 32	960	120.0	107.3 (6.6)	10.3 (2.3)	1.4 (0.6)	0.1 (0.2)	5.2 (0.7)
	25 × 30	750	93.8	108.2 (8.4)	10.6 (2.9)	2.2 (1.3)	0.1 (0.2)	7.1 (1.4)
	25 × 35	875	109.4	101.1 (11.5)	9.3 (3.9)	1.5 (0.8)	0.1 (0.2)	5.7 (1.3)
	30 × 30	900	112.5	101.8 (6.9)	9.2 (2.4)	1.7 (1.2)	0.1 (0.1)	6.0 (1.1)
	28 × 34	952	119.0	110.8 (9.4)	12.9 (3.8)	1.5 (1.3)	0.2 (0.5)	8.0 (1.6)
	30 × 35	1050	131.3	104.6 (11.6)	9.6 (4.4)	2.5 (2.1)	0.7 (1.3)	5.6 (1.5)

3 *vs* 3	18 × 25	450	75.0	120.8 (13.3)	14.2 (6.2)	2.4 (2.2)	0.1 (0.2)	12.9 (3.1)
	18 × 30	540	90.0	108.5 (8.3)	16.3 (5.3)	2.6 (2.1)	0.4 (0.6)	7.2 (1.9)
	18 × 32	576	96.0	109.2 (5.9)	11.2 (2.8)	1.4 (1.1)	0.2 (0.3)	10.1 (2.4)

The small-sided games are split for the number of players and pitch size (width × length). The total pitch area (m^2^) and area per player (m^2^ · player) have been calculated. The number of floaters, when required, is reported between bracket (i.e., one floater: +1f; two floaters: +2f). Average locomotor demands are reported for total distance (TD), high-speed running (HSR), very high-speed running (VHSR), sprint (SPR) and acceleration/Deceleration (Acc/Dec). Data are reported as mean (SD).

## SUPPLEMENTARY MATERIAL

**Title:** Small-sided games in elite youth soccer: area per player to replicate the match demands
